# Wireless facial biosensing system for monitoring facial palsy with flexible microneedle electrode arrays

**DOI:** 10.1038/s41746-024-01002-1

**Published:** 2024-01-15

**Authors:** Wenjianlong Zhou, Zhongyan Wang, Qin Xu, Xiangxiang Liu, Junshi Li, Huaiqiang Yu, Hui Qiao, Lirui Yang, Liangpeng Chen, Yuan Zhang, Zhe Huang, Yuxing Pang, Zhitong Zhang, Jiayan Zhang, Xiudong Guan, Shunchang Ma, Yingjie Ren, Xiaoyi Shi, Linhao Yuan, Deling Li, Dong Huang, Zhihong Li, Wang Jia

**Affiliations:** 1 Department of Neurosurgery, Beijing Tiantan Hospital, National Center for Neurological Disorders, Capital Medical University, 100070 Beijing, China; 2 School of Integrated Circuits, Peking University, 100871 Beijing, China; 3 Beijing Tongren Eye Center, Beijing Tongren Hospital, Capital Medical University, 100730 Beijing, China; 4 Sichuan Institute of Piezoelectric and Acousto-optic Technology, 400060 Chongqing, China; 5 Department of Neurophysiology, Beijing Neurosurgical Institute, Capital Medical University, 100070 Beijing, China; 6 China National Clinical Research Center for Neurological Diseases (NCRC-ND), 100070 Beijing, China; 7 Beijing Neurosurgical Institute, Capital Medical University, 100070 Beijing, China; 8 Beijing Advanced Innovation Center for Integrated Circuits, 100871 Beijing, China

**Keywords:** Biotechnology, Diseases of the nervous system

## Abstract

Facial palsy (FP) profoundly influences interpersonal communication and emotional expression, necessitating precise diagnostic and monitoring tools for optimal care. However, current electromyography (EMG) systems are limited by their bulky nature, complex setups, and dependence on skilled technicians. Here we report an innovative biosensing approach that utilizes a PEDOT:PSS-modified flexible microneedle electrode array (P-FMNEA) to overcome the limitations of existing EMG devices. Supple system-level mechanics ensure excellent conformality to the facial curvilinear regions, enabling the detection of targeted muscular ensemble movements for facial paralysis assessment. Moreover, our apparatus adeptly captures each electrical impulse in response to real-time direct nerve stimulation during neurosurgical procedures. The wireless conveyance of EMG signals to medical facilities via a server augments access to patient follow-up evaluation data, fostering prompt treatment suggestions and enabling the access of multiple facial EMG datasets during typical 6-month follow-ups. Furthermore, the device’s soft mechanics alleviate issues of spatial intricacy, diminish pain, and minimize soft tissue hematomas associated with traditional needle electrode positioning. This groundbreaking biosensing strategy has the potential to transform FP management by providing an efficient, user-friendly, and less invasive alternative to the prevailing EMG devices. This pioneering technology enables more informed decision-making in FP-management and therapeutic intervention.

## Introduction

Facial animation plays a crucial role in human communication as a primary medium for conveying emotions and nonverbal cues. However, facial palsy (FP) affects an estimated 118,000 individuals in the US^1,2^ and 100,000 in the UK^3^ annually. FP secondary to surgery has a reported incidence of 11–40%^4^, and tumors involving the facial nerves and adjacent tissues may compromise nerve function and result in persistent facial nerve paralysis^5–7^. Alterations in facial functionality and appearance due to FP, coupled with uncertainties surrounding recovery, contribute to anxiety, social withdrawal, and concealment of facial appearance as patients with FP often exhibit diminished self-esteem, heightened self-consciousness, and mood disorders^3^. Electrophysiological analyses of the facial nerves are vital for diagnosis, evaluation of lesion severity, and informed decision-making.

**Fig. 1:**
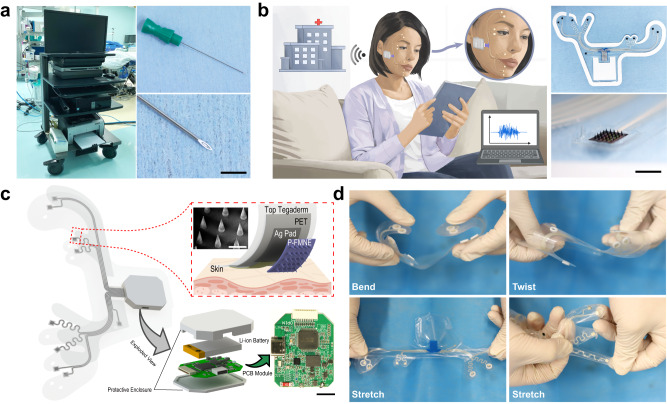
Advantages of Biomask system in comparison to traditional commercial electrophysiological monitoring equipment. **a** Photographic representation of customary commercial electrophysiological monitoring equipment and needle intramuscular electrodes (scale bar: 5 mm). **b** Photographic representation and schematic application of Biomask and P-FMNE (scale bar: 5 mm). **c** Exploded view schematic delineation of the essential mechanical and electrical constituents of the system (above/below scale bars: 500 μm/1 cm), and the red dashed box shows the structure of the electrode site area. **d** Biomask demonstrated in bent, twisted, and stretched geometries.

Electroneurography (ENoG) and electromyography (EMG) are the primary facial electrodiagnostic instruments for determining facial nerve and muscle functionality^8^. ENoG is effective during the initial phase of acute-onset facial paralysis within a window of 72 h to 21 days following lesion onset^9–11^. For long-standing facial paralysis (spanning weeks to months), EMG is more advantageous for detecting facial muscle activity and monitoring regeneration in cases in which reinnervation transpires^8^, for example, in FP secondary to neurosurgery. Nonetheless, existing EMG systems are sizable, costly, and unwieldy. Most platforms consist of data acquisition consoles connected to sensing electrode leads via numerous fixed electrical connections^12^. These platforms require expert technicians to position the needle electrodes, thereby hindering access to neurophysiological care in scenarios in which the required equipment or proficient technicians are lacking (Fig. 1a). Consequently, without extensive follow-up monitoring, treatment aimed at restoring satisfactory movement to the paralyzed face is seldom modified as a result of EMG data^13^.

Due to the required dermal penetration, needle electrodes utilized for EMG recordings often cause discomfort and/or soft tissue hematomas in patients^14,15^. Precise needle placement is critical for the integrity of the EMG signals. Furthermore, wired connections compound restrict electrode positioning and compound the spatial constraints. Silver/silver chloride (Ag/AgCl) gel electrodes, which inflict minimal harm, are widely employed to capture surface EMG signals. As facial musculature exhibits intricate geometric configurations and dynamic fluctuations, these Ag/AgCl gel electrodes are generally enveloped in expansive areas and robust adhesion materials (e.g., foam and nonwoven fabric) typically to ensure electrode–skin interface stability, resulting in interference due to traction and resistance during facial muscle contractions^16,17^.

In light of advancements in microfabrication techniques and material science, various lightweight, soft, thin-film electrodes have been developed and introduced to EMG research. However, their electrode–skin interface impedance (EII) remains relatively elevated, and the signal quality deteriorates caused by the stratum corneum barrier^18,19^. Microneedle electrodes, on the other hand, have diminutive dimensions and can penetrate the stratum corneum, establishing a low-impedance electrode–skin interface^20^. However, most microneedle electrode substrates are rigid and the adhesive schemes employed resemble those of gel electrodes, thus inhibiting the full realization of the benefits of microneedle electrodes^21^.

To surmount the limitations of contemporary EMG devices and electrodes^12,14,22–25^, we devise the “Biomask system,” a soft, conformal, wireless facial EMG signal acquisition system based on a PEDOT:PSS-modified flexible microneedle electrode (P-FMNE) array. We also present its evaluation in clinical trials, with this research representing the clinical study on facial paralysis monitoring using microneedle electrodes. In contrast to existing EMG systems, Biomask’s soft system-level mechanics facilitate intimate mechanical adherence to delicate curvilinear facial regions, enabling the capture of electrical activity (e.g., EMG) and targeted muscle group movements in response to real-time direct nerve stimulation during neurosurgical procedures. Furthermore, in this system, EMG signals can be wirelessly transmitted to hospitals via servers. This diminished technological barrier can enhance access to longitudinal patient follow-up data, enabling timely treatment recommendations (Fig. 1b, Supplementary Table 1).

## Results

### Overview of system design and fabrication

Modern advancements in material science, mechanical schematics, and fabrication techniques have paved the way for thin, mechanically flexible electronic frameworks capable of facilitating multimodal perception of the skin’s surface at virtually any body site. These platforms combine high-quality electronics and biosensors with wireless capabilities to monitor muscle responses to neural impulses and intraoperative stimulation with exceptional precision. The proposed Biomask system is one such platform, and Fig. 1c illustrates a deconstructed schematic of its design. P-FMNEs, designed according to the anatomical positions of facial muscles, are embedded in an ultra-thin, flexible patch resembling a “facial mask” that conformally covers the curved facial skin. This configuration enables patients to easily place the electrodes in the correct position without professional guidance. The electrodes are interconnected with a wireless transmission module located near the ear through slender serpentine wiring, facilitating wireless monitoring of facial EMG. The resulting form factor and intimate skin interface is a marked divergence from those of conventional wearable devices, which employ rigidly packaged electronic components affixed to the skin via straps, penetrating pins, foam, or nonwoven fabric. Stretching, twisting, bending, and other intricate modes of deformation do not disrupt the functionality of Biomask (Fig. 1d). The serpentine wire around the mouth has favorable mechanical and electrical properties and can accommodate the deformation of the skin. (Supplementary Fig. 1) Secure and comfortable adherence to the skin, even in tightly curved anatomical regions and sensitive body parts (e.g., the face), permits simultaneous EMG and motion sensing from multiple high-flexion or contractile muscle groups in a manner that is imperceptible to the patient and does not require guidance from a physician.

### Characterizations of PEDOT:PSS-modified flexible microneedle electrodes (P-FMNEs) and Biomask

High-performance bioelectrodes are the core element of future wearable devices. In terms of electrophysiological monitoring, microneedle electrodes can pierce the skin stratum corneum and exhibit excellent electrical properties compared to dry electrodes^26,27^. The P-FMNE substrate of the microneedles used for Biomask was fabricated using a micro-molding process that is compatible with flexible materials, and PEDOT:PSS was electrodeposited on its surface after double-sided sputtering of Ti/Au (Supplementary Figs. 2 and 3). Scanning electron microscope (SEM) images and photographs showed that PEDOT:PSS completely covered the microneedle surface (Supplementary Figs. 4, 6, and 8). The thickness of PEDOT:PSS was the highest at the tip of the microneedle, which was about 7.54 µm, but the thickness of PEDOT:PSS located under 450 µm of microneedle was relatively uniform, all around 2–3 µm, without significant variation in thickness (Supplementary Table 2). Microneedle electrodes with different heights (250–550 μm) were fabricated and their EII at the mentalis muscle was tested to determine the most appropriate microneedle height. With a microneedle height of 500 μm, the EII had a significant decrease, and the electrical characteristic of the P-FMNE was excellent, which was below 10 kΩ at a frequency of 10 Hz–10 kHz. The P-FMNE with a microneedle height of 500 μm was finally selected (Supplementary Fig. 5). The insertion force of the P-FMNE was about 0.35 N. Before the P-FMNE is inserted into the skin, the skin deformed by about 0.73 mm under the pressure of the tip of the P-FMNE (Supplementary Fig. 7).

**Fig. 2:**
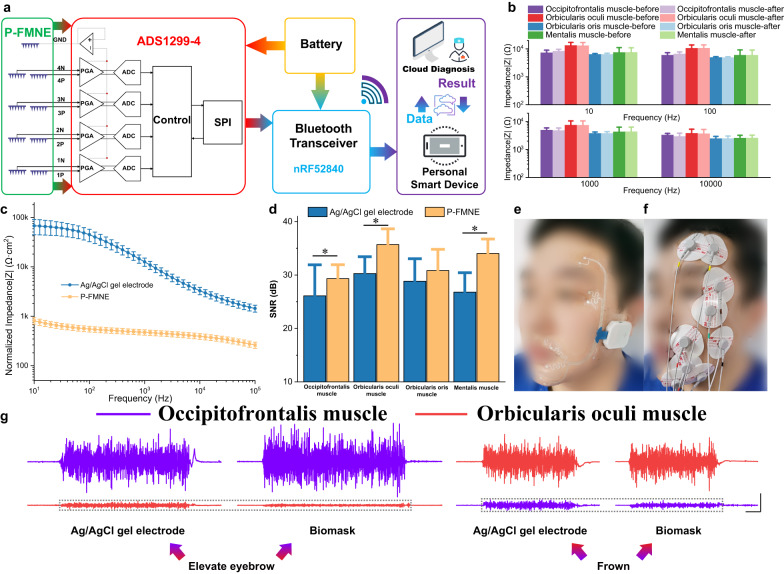
Wearable Biomask system with microelectrode arrays in a soft, stretchable design. **a** System architecture flow chart. **b** Electrode–skin interface impedance (EII) before and after comprehensive evaluation of facial muscles including the occipitofrontalis, orbicularis oculi, orbicularis oris, and mentalis muscles (*n* = 3). **c** Normalized EII (EII*area) of Ag/AgCl gel electrode and P-FMNE at the same position, Ag/AgCl gel electrode (*n* = 5) vs. P-FMNE (*n* = 5). **d** Signal-to-noise ratio (SNR) of Ag/AgCl gel electrodes and P-FMNE at occipitofrontalis, orbicularis oculi, orbicularis oris, and mentalis muscles. *P* values for comparing the SNR: Ag/AgCl gel electrode (*n* = 20) vs. P-FMNE (*n* = 20) of occipitofrontalis muscle in evaluating eyebrow, *P* = 0.029; Ag/AgCl gel electrode (*n* = 20) vs. P-FMNE (*n* = 20) of orbicularis oculi muscle in frowning, *P* < 0.001; Ag/AgCl gel electrode (*n* = 20) vs. P-FMNE (*n* = 20) of mentalis muscle in baring teeth smiling, *P* < 0.001. **e** Photograph of a patient wearing a Biomask with signal acquisition and wireless transmission circuit module inside the white box, and the identifiable photograph is fully consented by the written consent. **f** Photograph of a patient wearing traditional clinical Ag/AgCl gel electrodes, and the identifiable photograph is fully consented by the written consent. **g** EMG of Ag/AgCl gel electrodes and Biomask electrodes at occipitofrontalis muscle and orbicularis oculi muscles (left: elevate eyebrow; right: frown; vertical/horizontal scale bars: 250 μV/200 ms), and the gray dashed boxes facilitate the reader’s comparison of EMG signal amplitudes without special meaning. All error bars denote s.d. *P < 0.05; and an unpaired, two-tailed Student’s *t*-test was used for (**d**).

The P-FMNE was small and flexible (Supplementary Fig. 8a–f), and mechanically and electrically coupled to a flexible patch based on commercial transparent film dressings (3M Tegaderm, with Young’s modulus of ~7 kPa and a thickness of 47 μm) through a conductive silver paste. The P-FMNEs can conform to the curved facial skin where they are located, with the substrate bending inward or outward. The flowchart in Fig. 2a summarizes the system architecture and overall wireless operation of the Biomask system. The circuit module facilitates differential input of the EMG signal via the ADS1299-4 chip, amplifies the signal by 6× through a programmable gain amplifier (PGA), and conducts analog-to-digital conversion of the low-noise amplified signal. It samples the input signal at a rate of 500 Hz and enables the EMG signal to be input to the nRF52840-based Bluetooth transceiver module through an SPI interface. This Bluetooth transceiver module transmits the patient’s EMG signal to the user’s smartphone or tablet.

Preliminary evaluations of the system were executed with the Biomask worn on the facial surface of a 24-year-old male. The accuracy of the signals transmitted to the user was verified using a signal generator (Supplementary Fig. 9). There was nearly no variation observed for the EII before and after the comprehensive evaluation of facial muscles, and no significant increase in EII with up to 6 h of daily wear, demonstrating that the Biomask can guarantee tight electrode and skin conformation and exhibits sufficiently robust performance to accommodate postoperative FP assessment and intraoperative EMG monitoring (Fig. 2b and Supplementary Fig. 10).

As shown in Fig. 2c, d, we compared the P-FMNEs with traditional Ag/AgCl gel electrodes which were placed in the same position. the normalized impedance of the P-FMNEs at the mentalis muscle was significantly lower than that of Ag/AgCl gel electrodes (P-FMNEs: 0.47 kΩ*cm^2^@1 kHz and 0.81 kΩ*cm^2^@10 HZ; Ag/AgCl gel electrodes: 12.67 kΩ*cm^2^ @ 1 kHz and 67.92 kΩ*cm^2^@10 HZ), and the P-FMNEs exhibited higher signal-to-noise ratios (SNRs) at the four fast target muscles. We compared the Biomask electrodes with traditional Ag/AgCl gel electrodes. Biomask’s wireless acquisition scheme markedly improved wearing comfort and convenience compared to the cumbersome lead wires associated with traditional electrodes, and mitigated traction and resistance interference during facial muscle contractions (Fig. 2e, f and Supplementary Fig. 11). More significantly, owing to the small size of the P-FMNE (3 mm × 3 mm), the Biomask electrodes could be positioned more precisely on the targeted muscle surface. This can mitigate the signal interference between different muscles, thus enabling the EMG signal to be acquired with a higher spatial resolution when the patient elevates their eyebrow and frowns (Fig. 2g and Supplementary Fig. 12).

### Utilizing Biomask for comprehensive facial muscle monitoring

**Fig. 3:**
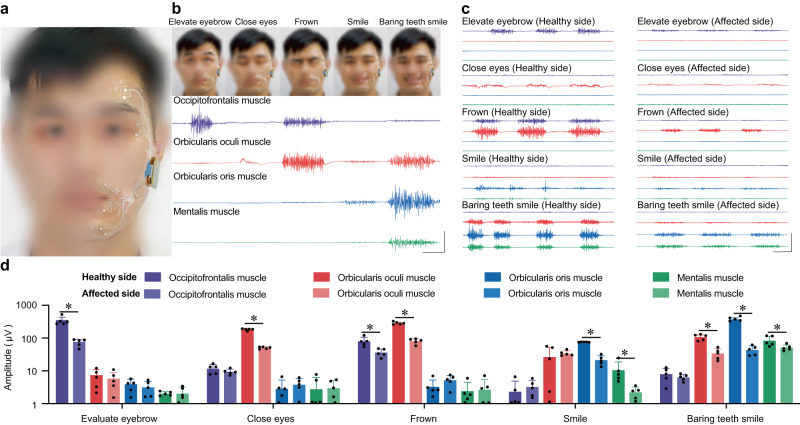
Biomask enables precise assessment of facial myoelectric activity during distinct expressions. **a** Photograph of Biomask donned by a subject. To ensure ease of application and unobstructed facial mobility, Biomask utilizes unilateral facial electromyography (EMG) surveillance, encompassing nine electrodes: four pairs allocated to the frontalis, orbicularis oculi, orbicularis oris, and mentalis muscles (where one of the pair is designated as the recording electrode and its counterpart is the reference electrode), with the final electrode situated on the cheek and functioning as a ground, and the identifiable photograph is fully consented by the written consent. **b** Biomask procures facial EMG data during specified expressions (elevate eyebrow, close eyes, frown, smile, smile with bared teeth), and the identifiable photograph is fully consented by the written consent (vertical/horizontal scale bars: 500 μV/200 ms). **c** Juxtaposition of facial electromyographic waveforms on the healthy and afflicted sides in patients with unilateral facial paralysis (vertical/horizontal scale bars: 1000 μV/400 ms). **d** Appraisal of facial EMG amplitude disparities between the healthy and compromised sides during particular expressions in individuals with unilateral facial paralysis. *P* values for comparing the EMG amplitudes: healthy side (*n* = 5) vs. affected side (*n* = 5) of occipitofrontalis muscle in evaluating eyebrow, *P* < 0.001; healthy side (*n* = 5) vs. affected side (*n* = 5) of orbicularis oculi muscle in closing eyes, *P* < 0.001; healthy side (*n* = 5) vs. affected side (*n* = 5) of occipitofrontalis muscle in frowning, *P* = 0.007; healthy side (*n* = 5) vs. affected side (*n* = 5) of orbicularis oculi muscle in frowning, *P* < 0.001; healthy side (*n* = 5) vs. affected side (*n* = 5) of orbicularis oris muscle in smiling, *P* < 0.001; healthy side (*n* = 5) vs. affected side (*n* = 5) of mentalis muscle in smiling, *P* = 0.044; healthy side (*n* = 5) vs. affected side (*n* = 5) of orbicularis oculi muscle in baring teeth smiling, *P* < 0.001; healthy side (*n* = 5) vs. affected side (*n* = 5) of orbicularis oris muscle in baring teeth smiling, *P* < 0.001; healthy side (*n* = 5) vs. affected side (*n* = 5) of mentalis muscle in baring teeth smiling, *P* = 0.046. All error bars denote s.d. *P < 0.05 and an unpaired, two-tailed Student’s *t*-test was used for (**d**).

The innovative Biomask system facilitates a comprehensive evaluation of facial muscle functionality by guiding patients through a series of five distinct actions. These actions, specifically chosen to target various aspects of facial muscle performance, encompass the movement of the eyebrows, closing of the eyes, frowning, smiling with concealed teeth, and smiling with bared teeth. The advanced biosensors integrated into the Biomask ensure accurate detection of EMG signal amplitudes, enabling a comprehensive analysis of facial muscle performance during each movement. In addition, motion artifacts may occur at the very beginning and end of facial movements. The motion artifacts are large relative to the EMG signal amplitude during the “close eyes”, and it is necessary to select the section of the EMG signal that is unaffected by the motion artifacts when evaluating the EMG signal. By assessing the amplitude variations, clinicians can accurately evaluate the patient’s facial muscle function and determine the severity of facial paralysis (Fig. 3a, b, Supplementary Figs. 13 and 14; Supplementary Video 1).

### Electromyography (EMG)-based facial palsy severity grading

In clinical investigations, the Biomask was positioned on the facial musculature innervated by the facial nerve of consenting patients with vestibular schwannomas (VSs) before, during, and following surgical interventions (Table 1, at the Department of Neurosurgery, Beijing Tiantan Hospital, China). Owing to inter-individual heterogeneity, the amplitudes of facial EMG vary between subjects. To counteract the data discrepancies caused by these disparities, we evaluated the severity of hemifacial paresis using contralateral facial EMG as a control. During specific movements, the Biomask acquired bilateral facial EMG signals from the patients with unilateral FP (Fig. 3c). After analyzing and comparing the data, we determined that the contractile capacity of facial muscles on the affected side was substantially lower than that on the healthy side (Fig. 3d). These electrophysiological findings provide quantitative support for stratifying FP severity as opposed to traditional classifications that rely primarily on clinicians’ subjective assessments.

**Table 1:** Summary of patient information.

Patient	Age	Gender	Diagnosis	Surgery	Monitored nerve	Recorded muscle
1	35	Male	Vestibular schwannoma (right)	Retrosigmoid approach tumor resection	7th cranial nerve (facial)	Facial muscle
2	43	Female	Vestibular schwannoma (right)	Retrosigmoid approach tumor resection	7th cranial nerve (facial)	Facial muscle
3	27	Female	Vestibular schwannoma (left)	Retrosigmoid approach tumor resection	7th cranial nerve (facial)	Facial muscle
4	29	Male	Vestibular schwannoma (right)	Retrosigmoid approach tumor resection	7th cranial nerve (facial)	Facial muscle
5	24	Female	Vestibular schwannoma (right)	Retrosigmoid approach tumor resection	7th cranial nerve (facial)	Facial muscle
6	36	Male	Vestibular schwannoma (left)	Retrosigmoid approach tumor resection	7th cranial nerve (facial)	Facial muscle

### Intraoperative monitoring of facial nerve–muscle function

Standard neurosurgical techniques for various interventions expose the facial nerve (e.g., resection of VSs). Direct nerve electrical stimulation is a prevalent method for locating and evaluating nerve–muscle interfaces. In this method, electrical pulses are used to stimulate nerves and induce action potentials and muscle contractions. The resulting EMG responses are recorded using needle electrodes and data acquisition systems. The stimulation current threshold, which is the minimum current required to evoke detectable EMG signals, is highly nonlinear. If the distances between the stimulation and measurement sites are known, it is possible to determine nerve conduction velocity, thereby identifying nerve damage. Although threshold levels fluctuate owing to physiological factors and the relative positions of nerves/muscles, comparing measured thresholds is an effective method for quantitatively comparing the skin-interfaced Biomask with other established electrophysiological monitoring devices.

**Fig. 4:**
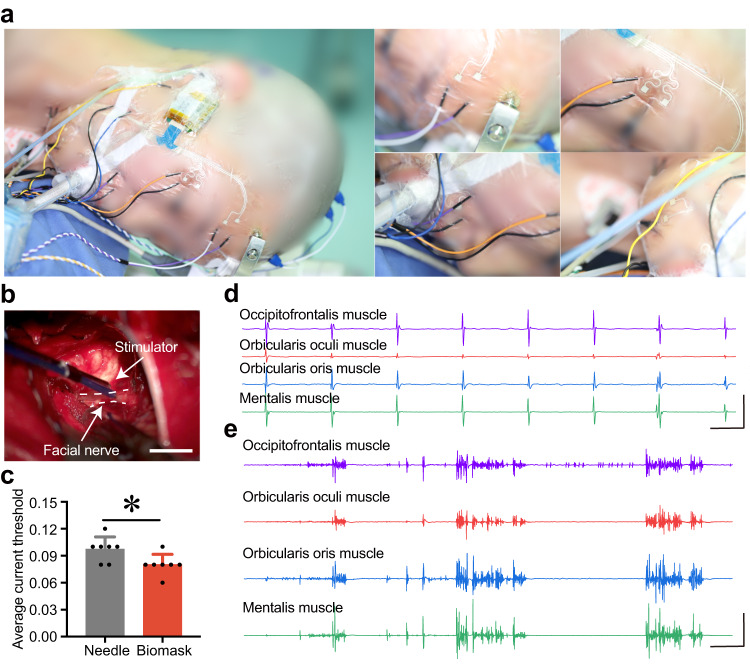
Comparative analysis of EMG recordings procured using Biomask and standard neurophysiological monitoring apparatus during vestibular schwannoma (VS) surgery. **a** Anatomical positioning of Biomask and needle electrodes on the face and the identifiable photograph is fully consented by the written consent. **b** Photograph illustrating stimulator employed for electrical stimulation of the facial nerve to assess neural function during VS surgery (scale bar: 1 cm), and the white dashed line is the outline of the facial nerve. **c** Comparison of stimulation current thresholds for the dual monitoring systems ascertained utilizing the configuration delineated in (**a**). *P* values for needle (*n* = 7) vs. Biomask (*n* = 7) in comparing the average current threshold, *P* = 0.046. **d** EMG waveforms elicited by stimulator-induced electrical stimulation captured using Biomask during VS surgery (vertical/horizontal scale bars: 100 μV/500 ms). **e** EMG waveforms induced by surgical manipulation and traction of the facial nerve acquired using Biomask during VS surgery (vertical/horizontal scale bars: 100 μV/500 ms). All error bars denote s.d. *P < 0.05; and an unpaired, two-tailed Student’s *t*-test was used for (**c**).

Here, we describe the Biomask clinical studies on consenting patients undergoing needle EMG recordings *during* VS surgery. Measurements from the wireless Biomask were compared to those from traditional needle EMG electrodes (cascade electronic intraoperative neurophysiological monitoring device, Cadwell) with hard-wired interfaces to conventional data acquisition systems (Fig. 4a). In most surgeries, both recording systems were placed near the surgical access point to the facial nerve for EMG capture during stimulation (Fig. 4b). Owing to the advanced microneedle electrode materials, the Biomask exhibited relatively lower stimulation current threshold levels compared to traditional intraoperative electrophysiological devices using needle electrodes, demonstrating the advantages of Biomask in EMG signal acquisition (Fig. 4c).

Biomask is thus not only adept at monitoring direct facial nerve electrical stimulation to identify and assess nerve–muscle interfaces (Fig. 4d, Supplementary Video 2) but also facilitates the monitoring of intraoperative passive tugging on the facial nerve induced by surgical manipulation (Fig. 4e, Supplementary Video 3), thereby minimizing the risk of nerve injury during neurosurgery.

### Enhancing facial paralysis management

**Fig. 5:**
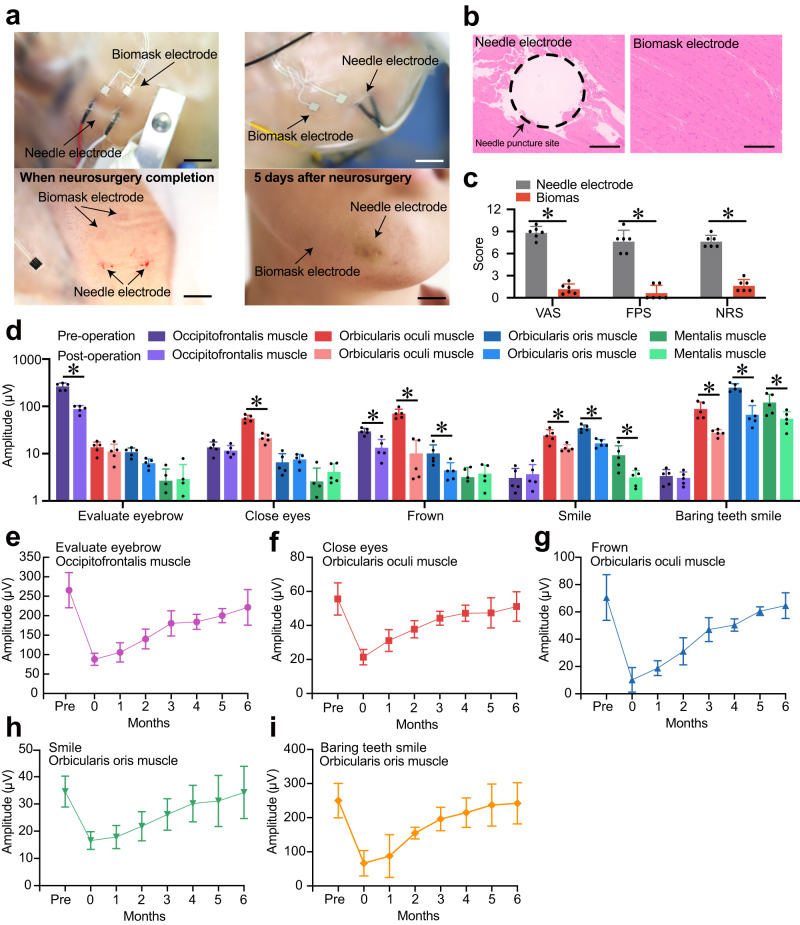
Biomask’s microelectrode arrays inflict no dermal trauma and are suitable for postoperative facial palsy monitoring in VS patients. **a** Photographic evidence illustrating hemorrhaging and ecchymosis resulting from electrophysiological monitoring with needle electrodes, particularly compared to microelectrode arrays, which demonstrably preserve skin integrity, and the identifiable photograph is fully consented by the written consent (scale bars: 1 cm). **b** Histopathological images depicting facial musculature damage induced by needle electrodes (scale bars: 200 μm), while microelectrode arrays remain innocuous (*n* = 3 mice for needle electrodes; *n* = 3 mice for Biomask electrodes), and the black circular dashed line is the needle puncture site. **c** Comparative analysis of patient pain scores during facial EMG surveillance employing needle electrodes and microelectrode arrays. *P* values for VAS: needle (*n* = 6) vs. Biomask (*n* = 6), *P* < 0.001. *P* values for FPS: needle (*n* = 6) vs. Biomask (*n* = 6), *P* < 0.001. *P* values for NRS: needle (*n* = 6) vs. Biomask (*n* = 6), *P* < 0.001. **d** Assessment of facial EMG amplitude differentials between preoperative and postoperative stages during distinct expressions in individuals afflicted with unilateral VS. *P* values for comparing the EMG amplitudes: pre-operation (*n* = 5) vs. post-operation (*n* = 5) of occipitofrontalis muscle in evaluating eyebrow, *P* < 0.001; healthy side (*n* = 5) vs. affected side (*n* = 5) of orbicularis oculi muscle in closing eyes, *P* < 0.001; healthy side (*n* = 5) vs. affected side (*n* = 5) of occipitofrontalis muscle in frowning, *P* = 0.002; healthy side (*n* = 5) vs. affected side (*n* = 5) of orbicularis oculi muscle in frowning, *P* < 0.001; healthy side (*n* = 5) vs. affected side (*n* = 5) of orbicularis oris muscle in frowning, *P* = 0.048; healthy side (*n* = 5) vs. affected side (*n* = 5) of orbicularis oculi muscle in smiling, *P* = 0.015; healthy side (*n* = 5) vs. affected side (*n* = 5) of orbicularis oris muscle in smiling, *P* < 0.001; healthy side (*n* = 5) vs. affected side (*n* = 5) of mentalis muscle in smiling, *P* = 0.031; healthy side (*n* = 5) vs. affected side (*n* = 5) of orbicularis oculi muscle in baring teeth smiling, *P* = 0.005; healthy side (*n* = 5) vs. affected side (*n* = 5) of orbicularis oris muscle in baring teeth smiling, *P* < 0.001; healthy side (*n* = 5) vs. affected side (*n* = 5) of mentalis muscle in baring teeth smiling, *P* = 0.043. **e** EMG variations in the compromised frontalis muscle during eyebrow motion appraisal in follow-up examinations for patients diagnosed with unilateral VS. **f–i** EMG variations in the compromised orbicularis oculi muscle during appraisal of eye-closing, frowning, smiling, and smiling-with-bared-teeth motions, respectively, in follow-up examinations for patients diagnosed with unilateral VS. All error bars denote s.d. *P < 0.05; an unpaired, two-tailed Student’s *t*-test was used for (**c** and **d**).

Biomask, which features innovative microneedle electrodes, provides considerable advantages over traditional needle electrodes in terms of patient comfort and skin preservation. These tiny advanced material electrodes penetrate the stratum corneum of the skin with minimal disruption, effectively reducing the risk of skin damage. In contrast to conventional needle electrodes, which pierce the skin and muscle and cause bleeding upon removal, in the above clinical trials, Biomask electrodes left only minor traces on the skin without inducing soft tissue hematomas. A few days after the procedure, noticeable bruising remained on the skin punctured by the needle electrodes, whereas the skin that had been in contact with the Biomask electrodes showed no signs of damage (Fig. 5a).

Additionally, animal experiments simulating skin injury and subsequent hematoxylin and eosin (HE) staining revealed that traditional needle electrodes caused significant muscle damage in test subjects, while Biomask’s microneedle electrodes did not harm the skin or muscles (Fig. 5b). This non-invasive approach not only minimizes the risk of soft tissue hematomas and other complications associated with conventional needle electrode placement but also alleviates pain and discomfort during the EMG recording process (Fig. 5c, Supplementary Figs. 15 and 16).

Owing to its exceptional biocompatibility with facial skin, as demonstrated above, we conducted further experiments using Biomask to monitor EMG in patients with facial paralysis. By collecting preoperative and postoperative facial EMG data from the affected side of VS patients (Fig. 5d, Supplementary Videos 4 and 5), we evaluated the severity of postoperative FP and provided electrophysiological evidence for the grading of facial paralysis.

The integrated face mask design was conceptualized to facilitate patients in achieving precise electrode positioning without expert guidance, requiring instruction only before the initial use (Supplementary Fig. 17). Using Biomask’s wireless architecture, data were transmitted via cloud services, and we succeeded in accessing multiple facial EMG datasets during the 6-month follow-up (Fig. 5e–i). Data analysis revealed a pattern in the timeline of facial nerve function recovery in patients with VS who underwent surgical treatment.

## Discussion

Current conventional clinical facial nerve electro-diagnostics are limited in terms of size, complexity, and portability. The findings reproduced herein demonstrate the viability of the skin-mounted wireless Biomask for uninterrupted electrical and mechanical surveillance of facial muscle movements in intraoperative and postoperative clinical contexts. Its ultra-flexible, miniaturized form factor and precise biosensors facilitate direct adherence to delicate curvilinear facial regions, and its exceptional stretchability and skin conformability enable the electrodes to fit seamlessly on the facial skin, capturing every EMG and motion signal during a variety of facial movements. The employment of the P-FMNE guarantees consistent recordings with an optimal SNR while simultaneously diminishing the electrode size compared to traditional surface electrodes. This remote, wireless, and user-friendly monitoring apparatus promotes long-term patient follow-up, empowering clinicians to appraise the effectiveness of therapy based on signal alterations and adapt treatment strategies accordingly.

Contemporary advancements in electronic technology have facilitated high-precision signal recording and long-term data storage; however, the interface between electronic systems and the body remains a primary challenge for bio-signal acquisition systems. We compared the performance of Biomask electrodes with that of conventional commercial electrodes based on the impedance characteristics and the quality of the obtained EMG signals. The Biomask electrode operates on the premise that microneedles can capture signals by penetrating the non-conductive stratum corneum and accessing the viable epidermis^27–30^. Across the tested frequency range, the Biomask electrode consistently exhibited lower electrode–skin interface resistance and overall impedance than the Ag/AgCl gel electrode. The SNR results demonstrated that the electrical characteristics of the Biomask electrodes surpassed those of the Ag/AgCl gel electrode, confirming that Biomask’s electrode channels possessed an adequately low impedance for measuring EMG activity.

Furthermore, in contrast to conventional needle electrodes, the Biomask electrodes, coated with PEDOT:PSS^31,32^, enable reduced motor stimulation thresholds. Moreover, none of the participants reported pain, bleeding, inflammation, or any discernible discomfort associated with the use of Biomask electrodes during or after the studies. These findings exemplify Biomask’s ability to mitigate electrode–tissue interface impedance, decrease motor stimulation thresholds, augment SNR, and maintain minimal invasiveness.

Electrophysiological diagnostics facilitate the evaluation of facial nerve and muscle functionality. In comparison to established intraoperative monitoring systems (Cadwell Cascade), Biomask’s physical design presents substantial advantages in electrophysiology, size, weight, and comfort without compromising signal fidelity or threshold detectability during intraoperative monitoring. This ease of use obviates the need for technicians who are specialized in needle electrode placement and EMG recording. These usability features, combined with the system’s wireless functionality, have the potential to significantly reduce clinical preparation time and effort in the operating room^14,33–35^. Specifically, the burden of needle electrode weight, tissue insertion complexity, and wired cable management—secured to the patient’s body and routed across the operating table to adapter boxes and computer control systems—could be considerably alleviated. In clinical trials, the EII of Biomask remained almost consistent during 6 h of wear, demonstrating its monitoring stability throughout neurosurgical procedures. Furthermore, the ease of setup offers a substantial advantage to medical institutions that lack trained personnel, thereby substantially enhancing the availability of preoperative and postoperative monitoring for facial nerve functional assessment. In hospitals where such intricate and expensive monitoring equipment is inaccessible or impractical, these appealing physical and operational attributes can encourage system adoption.

Currently, outcome tracking in FP primarily comprises patient-reported outcome measures and clinician-graded scoring systems. The absence of tangible and objective indicators hinders clinicians from routinely using such tracking systems. To address these limitations, the Biomask device was designed for portability, unobtrusiveness, and wireless data transmission to smart devices. This ease of setup provides significant benefits to untrained patients, enabling discharged patients to self-test at home while their EMG signals are transmitted to mobile terminals and remotely monitored by physicians. Physicians can swiftly assess data on the extent of a patient’s FP, evaluate the efficacy of current treatment procedures, and modify the treatment accordingly. Furthermore, since EMG is known to be most valuable within the timeframe of 2–3 weeks to 3 months following the onset of facial nerve injury, frequent, long-term monitoring of EMG in FP patients using Biomask presents a crucial electrodiagnostic tool for observing spontaneous nerve regeneration. During the postoperative period, patients do not need to wear the Biomask continuously; a few minutes during each follow-up check is adequate. The integrated design of the Biomask guarantees precise electrode placement while being worn by patients.

Essentially, needle EMG monitoring is conducted by inserting a conventional needle into the facial muscles of a patient and instructing the patient to perform designated tasks. With conventional needle electrodes, this frequently results in pain and/or soft tissue hematomas. In contrast, our Biomask was engineered for efficient and painless penetration into elastic human skin using arrays of minimally invasive (~500 μm) microneedles. Biomask’s minimal invasiveness was supported by patient reports immediately following surgery and after the passage of several days. Painless monitoring substantially enhances patient compliance and enables long-term follow-up.

FP imposes considerable functional and psychosocial challenges on affected individuals. The materials and device configurations presented herein for Biomask lay the groundwork for skin-mounted electronic sensing technologies and offer substantial capabilities for long-term facial nerve monitoring. As evidenced by our clinical studies, Biomask’s design facilitates stable, high-fidelity signal acquisition for functional assessment of the facial nerve in discharged patients. Moreover, the integration of Biomask measurement platforms with traditional standalone nerve stimulators presents surgeons with the opportunity to monitor facial nerve and muscle function during a broad array of operations where nerves are at risk of damage. Embracing such wireless wearable technology may not only simplify intraoperative monitoring but also improve patient outcomes through swift intervention by cloud-monitoring clinicians.

## Methods

### Clinical study design

This study was approved by the Beijing Tiantan Hospital, Capital Medical University Investigational Review Board, and informed consent was obtained from all patients before surgery and research participation (HX-B-2022025; May 27, 2022). This study was also approved by the Chinese Clinical Trial Registry (ChiCTR) with the trial registration number (ChiCTR2300072887; June 27, 2023).

Consecutive six VS patients were recruited at Beijing Tiantan Hospital, Capital Medical University for a prospective study of clinical validation of the Biomask system. To assess facial paralysis, patients were pretrained to accurately execute a series of five distinct actions targeting various aspects of facial musculature function, including eyebrow elevation, eye closure, furrowing the brow, smiling, and smiling with the teeth exposed. Prior to the Biomask application, the facial cleanliness of patients was ensured, and traditional electrodes were positioned according to guidelines at appropriate anatomical sites and performed the specified actions, repeating each three times. The EMG data from distinct facial muscles during these diverse actions were collected for subsequent analyses. For intraoperative neurophysiological monitoring, patients were administered with general anesthesia devoid of long-lasting paralytic agents, thus permitting muscle activity monitoring. Following anesthetic administration, the Biomask was placed on the facial skin overlying the pertinent muscles of the patient and concurrently monitored using standard needle EMG techniques. Nerve–muscle activity was recorded once the target nerves were surgically exposed, and direct current stimulation was applied under the guidance and supervision of the lead surgeon. All the identifiable photographs are fully consented to by written consent.

### Biomask fabrication

The Biomask system comprises four components: P-FMNE arrays (P-FMNEAs), a flexible patch, a wireless transmission module, and an intelligent device terminal.

The P-FMNE was constructed using microelectronic mechanical systems (MEMS) microfabrication techniques, as shown in Fig. S2. Multiple P-FMNEs are assembled onto a flexible patch to constitute P-FMNEAs.

1. Steel needles were assembled on a bare PCB board with through-holes, with a specialized mold securing the microneedle tips and ensuring a uniform height, while the reverse side was stabilized with epoxy resin.
2. A fabricated rigid mold was situated in a petri dish, filled with a 10:1 PDMS (polydimethylsiloxane, SYLGARD™ 184), and heated at 80 °C for 2 h to cure.
3. The PDMS mold was carefully removed from the rigid mold and placed in an oven at 120 °C for 5 h to achieve complete curing.
4. The PDMS mold’s surface was coated with polyimide precursor (POME, Sci-Tech Co., Ltd), heated on a hot plate at 100 °C for 2 h to vaporize the solvent, and then heated at 240 °C for 5 h to finalize imidization.
5. The polyimide film was delicately peeled off the PDMS mold.
6. Following an organic cleaning procedure, dual-sided sputtering of Ti/Au (20 nm/200 nm) was performed to achieve metallization.
7. Within an EDOT (3,4-ethylenedioxythiophene, 0.01 M; Sigma-Aldrich) and PSS (polystyrene sulfonate, 2.5 wt%; Sigma-Aldrich) solution, a Pt mesh electrode was positioned on the negative side of the assembly, with the microneedle electrodes on the positive side, facilitating the electrodeposition of PEDOT:PSS (30 μA for 300 s, 120 μA for 1800 s, and 160 μA for 40 min) via an electrochemical workstation (Multi Autolab/M101; Metrohm).
8. Finally, the complete P-FMNE was removed from the solution and cleaned in deionized water.

The flexible patch comprised a three-layer architecture: (1) the bottom 3M Tegaderm layer, principally designed to adhere the Biomask to the skin, ensuring conformal contact across varying facial expressions; (2) the central flexible PET (polyethylene terephthalate) circuit board, which processes metal wiring via screen printing; and (3) the top 3M Tegaderm layer, which encapsulates the PET circuit board to mitigate environmental interference. Both the bottom and top 3M Tegaderm layers incorporated release films to facilitate convenient self-application for patients. The flexible patch was fabricated by Dongguan Kentai Medical Supplies Co., Ltd. Initially, a plane die-cutting machine shaped the bottom 3M Tegaderm layer, followed by the alignment and attachment of a PET flexible circuit board (equipped with a double-sided adhesive) to the bottom 3M Tegaderm using a secondary projector. Subsequently, the top 3M Tegaderm layer was adhered to the flexible PET circuit board.

Measuring 40 × 50 × 10 mm^3^, the wireless transmission module primarily consisted of three elements: a 3D-printed shell, a lithium-ion battery, and a low-power circuit board. The 3D-printed shell was fixed to the flexible patch using a double-sided adhesive. High-quality EMG signals were transmitted to the intelligent device terminal via the wireless transmission system, and an application tailored to the wireless transmission module was installed to enable the real-time display and storage of EMG signals.

### Characterized the tensile and electrical properties of the serpentine wire

We fixed both ends of the serpentine wire on the mechanical testing machine by fixtures in the mechanical testing machine (ESM303, Mark-10) and measured the resistance of the serpentine wire during strain by the source meter (Keithley 2450). For measuring the maximum strain, the mechanical testing machine was set to move upward at a constant speed until the serpentine wire broke. To measure the cyclic strain, the cyclic parameters were set by the mechanical testing machine. The cycle number was 50, the travel was 60% strain (15 mm) and the time period was about 13 s.

### Measuring the thickness of PEDOT: PSS

The P-FMNE was placed in a cylindrical silicone container and secured by a fixture. Epoxy resin was poured into the cylindrical container and cured for 3 h at room temperature. Afterward, the cured cylindrical mold was removed from the container, and the mold was polished with 600-mesh sandpaper until the root of the P-FMNE was exposed. The mold was then polished sequentially with 1500-mesh, 3000-mesh, and 5000-mesh sandpapers to expose the entire cross-section of the P-FMNE. After organic cleaning, the mold was placed in the ESEM for observation.

### Conventional EMG sensing electrodes and stimulation

Needle electrodes (Rhythmlink, 13-mm-long, 0.4 mm in diameter, with a 1.5-m-long leadwire, SP119022, stainless steel) served to monitor nerve activity during the surgeries. A stimulation probe delivered direct current to the target site (Prass Standard Flush-Tip Probe, Medtronic Xomed, ~10 cm long, stainless steel with a plastic handle). The electrodes were electrically connected to an external stimulator box (conventional intraoperative monitoring system). The stimulation pulses consisted of monophasic waveforms (2.6 Hz, 200 μs pulse width) at adjustable current levels with a control resolution of 0.01 µA.

### Stimulation current threshold study

To determine the stimulation current thresholds, direct electrical stimulation was applied to the surface of the facial nerve. The stimulus commenced at subthreshold electrical current levels, (insufficient to elicit activity in the targeted muscle, as assessed using a standard EMG monitoring system). Subsequently, the stimulus intensity was incrementally escalated until a discernible muscle response was acquired by both the Biomask and conventional intraoperative monitoring systems independently. A proficient intraoperative monitoring specialist ascertained the threshold current value at the juncture when the EMG signal waveform was visually distinguishable. This procedure was repeated five times and the stimulus threshold level required to evoke a detectable muscle response was documented during each trial. The collected data were analyzed postoperatively. The reported values represent the mean of five trials, with error bars representing one standard deviation.

### Impedance characterization

The impedance of the P-FMNEs and Ag/AgCl gel electrode was measured with an electrochemical workstation (Multi Autolab/M101; Metrohm). We adopted a two-electrode system with electrodes fixed to the skin surface and applied a sine wave with a root-mean-square (RMS) amplitude of 0.35 V. The applied frequency ranged from 10 Hz to 100 kHz. The normalized impedance was calculated as follows:

$${{|Z|}}_{{\rm{normalized}}}={{|Z|}}_{{\rm{measured}}}\times {{{\rm {Area}}}}_{{{\rm {contact}}}}$$

### EMG signal acquisition and analysis

Regarding signal processing, EMG signals from the Biomask underwent second-order Butterworth band-pass filtering at 20–249 Hz to eliminate low-frequency noise. Signal segments were obtained as the 1000-ms-long data series from the middle of each high-amplitude EMG waveform triggered by muscle activity, while the noise segments were taken from the 1000-ms-long data series in the middle of the ‘relaxing’ area between two EMG waveforms. The RMS and SNR are calculated as follows:

$${{\rm {RMS}}}=\sqrt{\frac{1}{N}{\sum }_{n=1}^{N}{V}_{n}^{2}}$$

$${\rm{SNR}}=20{\log }_{10}\frac{{{{\rm {RMS}}}}_{{{\rm {signal}}}}}{{{{\rm {RMS}}}}_{{{\rm {noise}}}}}$$

Data processing was performed using Origin Pro software, ensuring a comprehensive and precise analysis of EMG signals procured from both conventional and Biomask electrodes. This sophisticated approach markedly enhances the appraisal of neuromuscular activity, ultimately contributing to optimized patient management and improved treatment outcomes for facial paralysis.

### Animal experiments

All animal procedures were performed in accordance with the guidelines of the Public Health Service Policy on Humane Care of Laboratory Animals and approved by the Institutional Animal Care and Use Committee of Beijing Tiantan Hospital, Capital Medical University (202101001). 6–8 weeks old BALB/c Nude mice raised in a Specific Pathogen Free (SPF) environment were used in the study.

### Electrode-induced muscle damage

To assess muscle damage caused by different types of electrodes, mice were anesthetized with 2% isoflurane in balanced oxygen, and then randomly categorized into two groups to simulate muscle injury. Under sterile conditions with external body warming, traditional needle electrodes were compared to innovative Biomask microneedle electrodes in terms of their effects on muscle. After the procedure, tissue samples were fixed in formalin, embedded in paraffin, and sectioned for histological examination. Subsequently, these samples were stained with Hematoxylin and Eosin (HE). The mice were euthanized with carbon dioxide gas immediately after the experiment.

### Insertion test of the P-FMNE

6–8 week-old BALB/c Nude mice were anesthetized with 2% isoflurane in balanced oxygen. Under sterile conditions with external body warming, we positioned the mice on a flat test bench. The flexible microneedle electrode (P-FMNE) was then gently placed on the mice’s skin surface, aligning the tip of the P-FMNE downwards. Pressure application was conducted using a mechanical testing machine (ESM303, Mark-10). Once the mechanical probe of the testing machine made contact with the P-FMNE, the accompanying electrode started moving downwards at a constant rate, correlating with the probe’s movement (Supplementary Fig. 7). The mice were euthanized with carbon dioxide gas immediately after the experiment.

### Statistical analysis

OriginPro 2022 (SR1 v.9.9.0.225), GraphPad (Prism 8.4.3), and Microsoft Excel 2016 (v.1807, 10325.20082) were used for all statistical analyses. All replicate numbers, error bars, *P* values, and statistical tests are indicated in the figure legends.

### Reporting summary

Further information on the research design is available in the Nature Research Reporting Summary linked to this article.

### Supplementary information

Supplementary Information

nr-reporting-summary

Supplementary Video 1

Supplementary Video 2

Supplementary Video 3

Supplementary Video 4

Supplementary Video 5

## Data Availability

All data supporting the findings of this study are available within the paper and its Supplementary Information.
