# Supplementary material for: Wireless facial biosensing system for monitoring facial palsy with flexible microneedle electrode arrays
Source: NPJ Digit Med. 2024 Jan 15;7:13. doi: 10.1038/s41746-024-01002-1 (PMC10789865; doi:10.1038/s41746-024-01002-1)
Supplement: Supplementary file 1 — Supplementary Information [file 41746_2024_1002_MOESM1_ESM.pdf]

Supplementary Table 1 | Comparison of our work versus literature reports on EMG monitoring[22-27](Y: Yes, N: No).

Reference	Form factor	Microneedle (Y/N)	Soft and stretchable electrode (Y/N)	Wireless (Y/N)	Facial muscles (Y/N)	Target region
22	The electrodes were screen printed using a conductive carbon ink	N	Y	Y	Y	The orbicularis oculi, the zygomaticus major, and the levator labii superioris muscles
23	A wired device using sets of pre-gelled, self-adhesive 0.7-cm Ag/AgCl electrodes with 1.5-cm inter-electrode spacing	N	N	Y	Y	The corrugator supercilii and zygomatic major muscles
24	A set of dry, arrayed sEMG sensors	N	N	Y	Y	The orbicularis oculi, the zygomaticus major, and the corrugator muscles
25	The conducting polymer PEDOT/PSS was modifed on the gold surface of the Polyimide-based fexible microneedle array (PI-MNA) electrodes	Y	Y	Y	N	The extensor digitorum, biceps, and tibialis anterior
26	Metal monopolar needle electrodes	N	Y	Y	Y	Frontalis, orbicularis oculi, and orbicularis oris muscles
27	hydrogel adhesive electrodes with Ag/AgCl backing layer； serpentine interconnect structures	N	N	N	N	Tibialis anterior muscle, foot muscle, trapezius muscle, deltoid muscle, finger extensor digitorum muscle, peroneus longus muscle
Our work	The conducting polymer PEDOT/PSS was modifed on the gold surface of the Polyimide-based fexible microneedle array (PI-MNA) electrodes	Y	Y	Y	Y	Facial muscle： Occipitofrontalis muscle， Orbicularis oculi muscle， Orbicularis oris muscie and Mentalis muscle

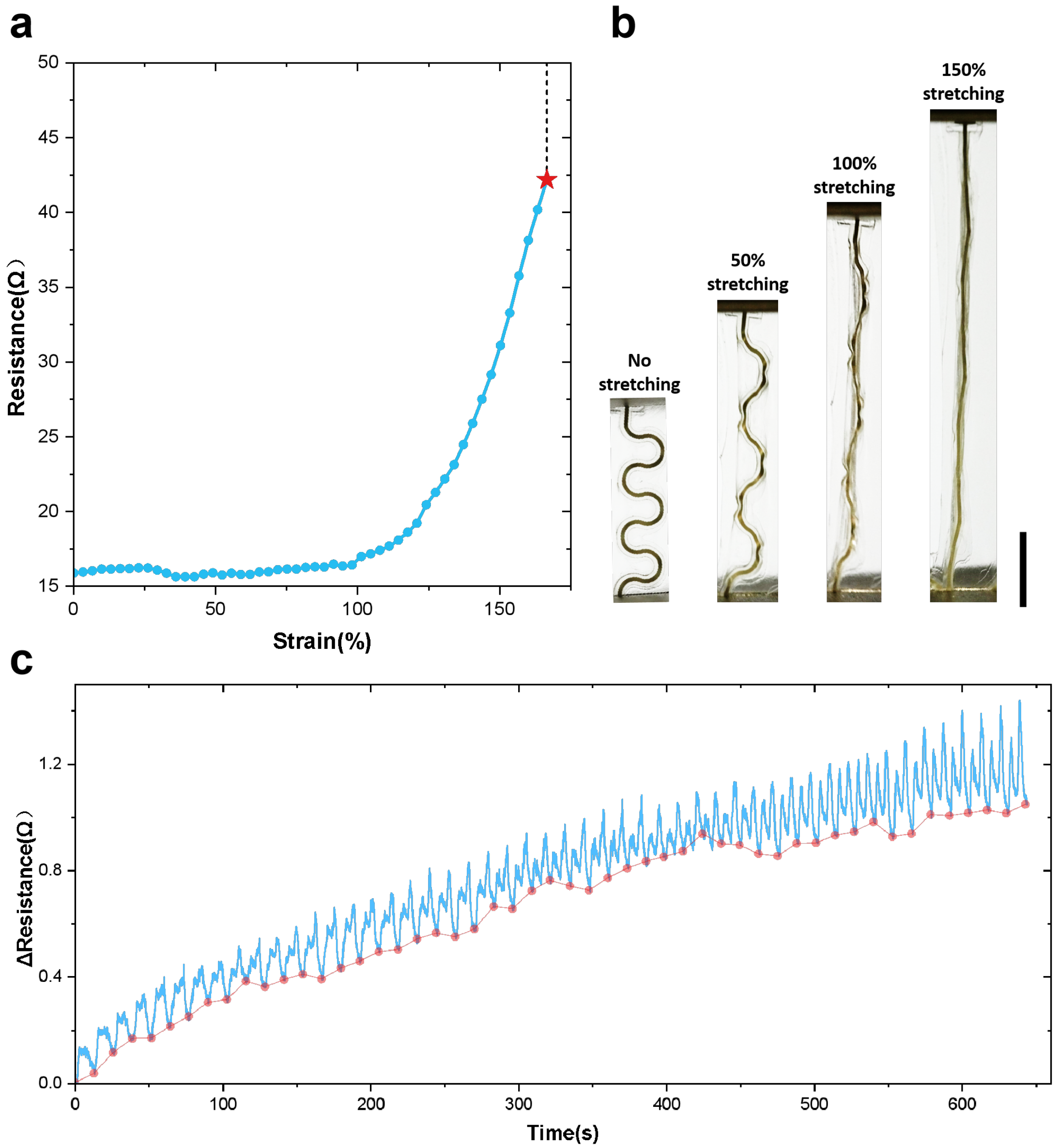

Supplementary Fig. 1 | Quantitatively characterized the tensile and electrical properties of the serpentine wire. a, resistance of the serpentine wire when gradually stretched, the red five-pointed star is the failure point of the serpentine wire, the black dashed line indicates infinite resistance after failure. **b,** photographs of serpentine wires at different stretching levels (scale bar: 10 mm). **c,** resistance change of the serpentine wire with 60% strain for 50 cycles, the red point is the starting or ending point of each cycle.

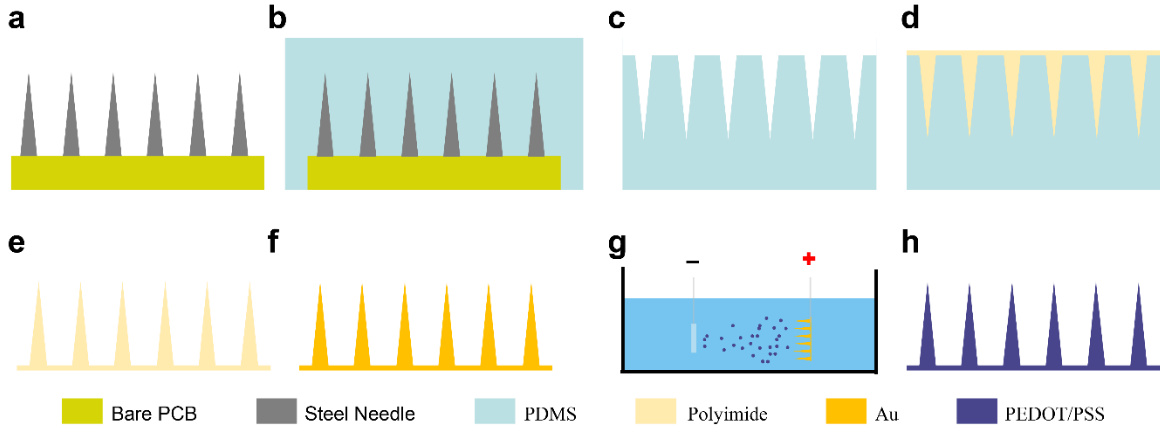

Supplementary Fig. 2 | Fabrication process of PEDOT:PSS-modified flexible microneedle electrode (P-FMNE). a, rigid mold. **b,** PDMS pouring and heat application. **c,** peeling off PDMS mold. **d,** pouring of polyimide precursor and heat application. **e,** peeling off polyimide film. **f,** double-sided sputtering of Ti/Au. **g,** electrodeposition of PEDOT:PSS. **h,** fabricated P-FMNE.

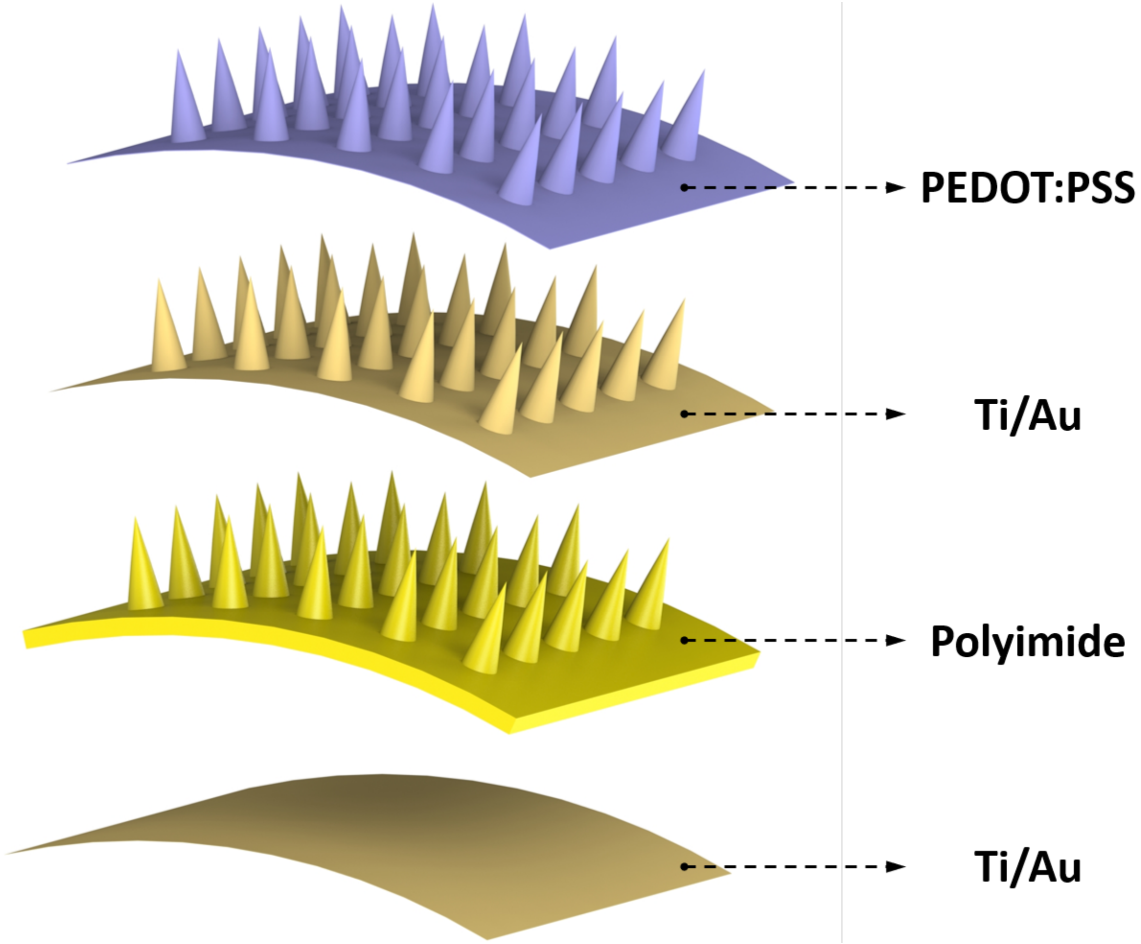

Supplementary Fig. 3 | Exploded view of the P-FMNE.

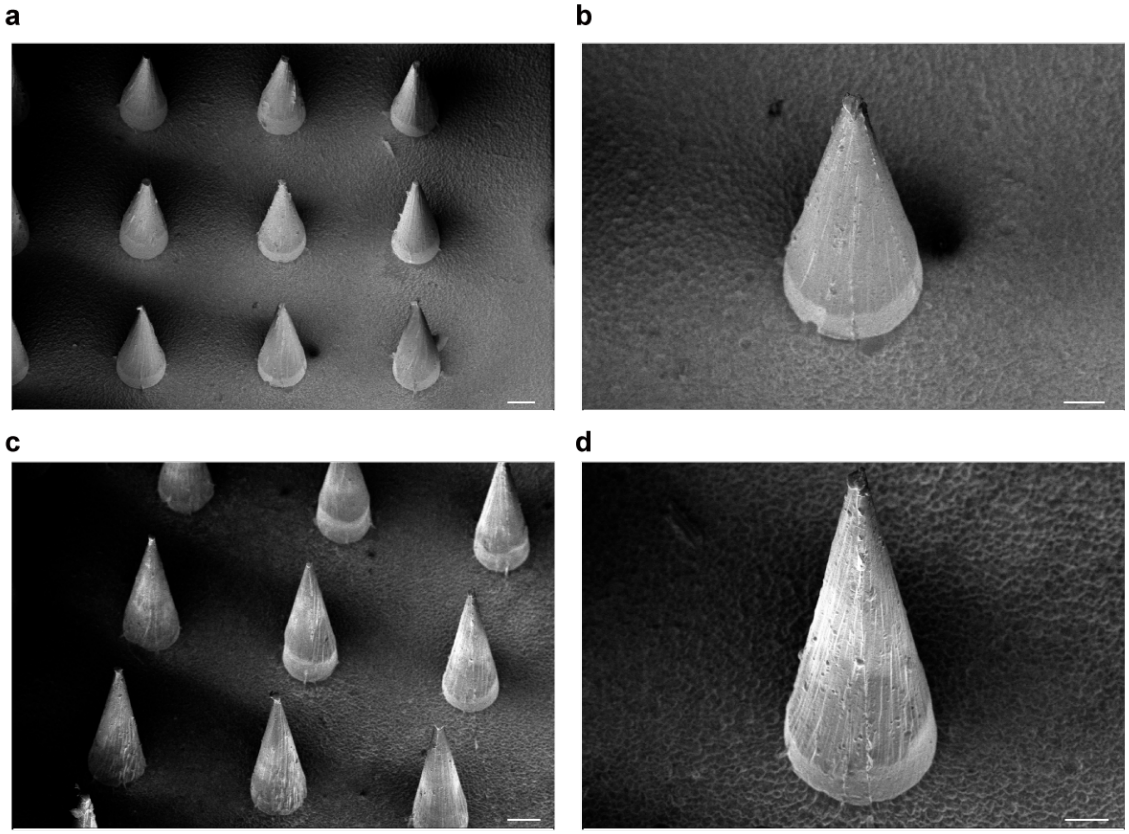

Supplementary Fig. 4 | Scanning electron microscope images of P-FMNE. a, P-FMNE after sputtering of Ti/Au (scale bar: 100 μm). **b,** single P-FMNE after sputtering Ti/Au (scale bar: 50 μm). **c,** P-FMNE after electrodepositing PEDOT:PSS (scale bar: 100 μm). **d,** single P-FMNE after electrodepositing PEDOT:PSS (scale bar: 50 μm).

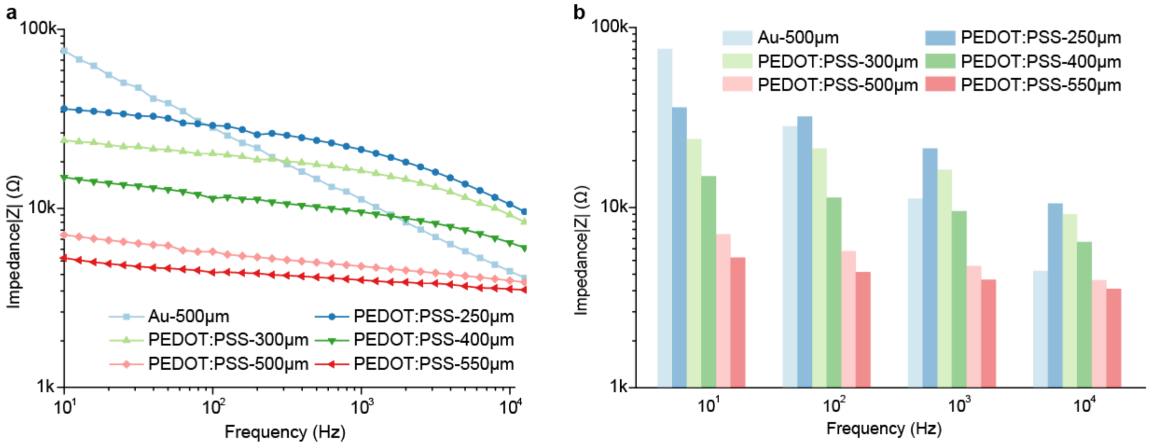

Supplementary Fig. 5 | Electrode– skin interface impedance (EII) for different microneedle heights. a, EII for different microneedle heights at 10–10000 Hz. **b,** EII for different microneedle heights at 10, 100, 1000 and 10000 Hz.

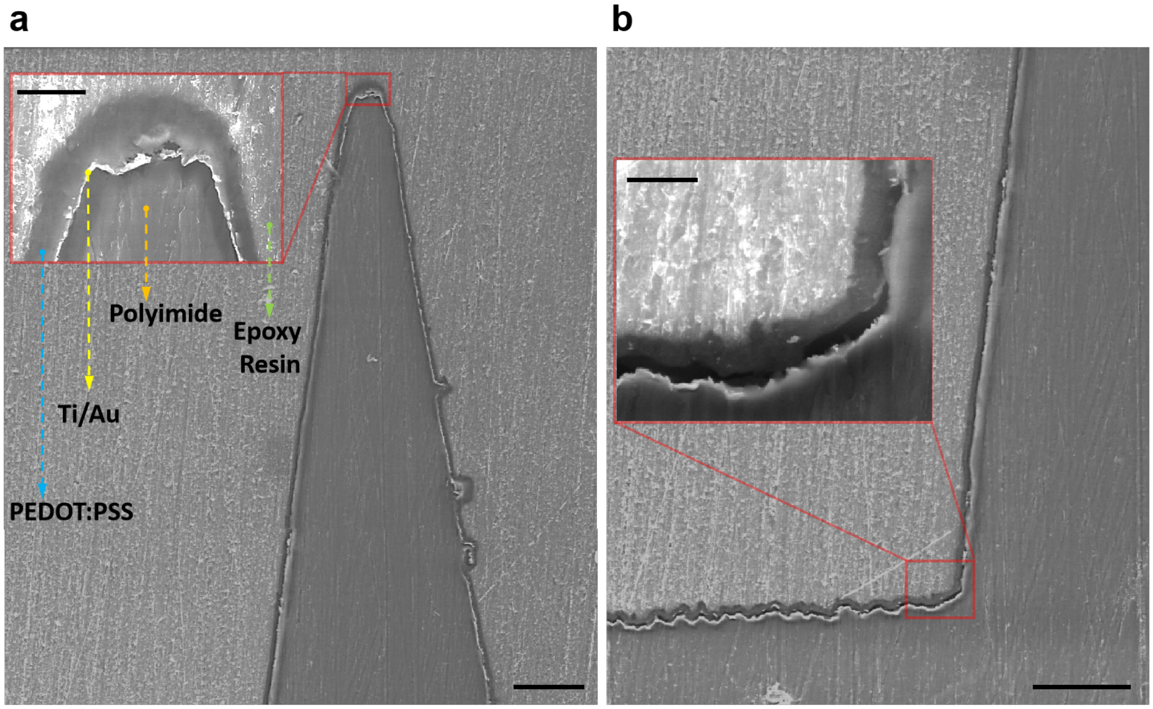

Supplementary Fig. 6 | Environmental scanning electron microscope images of the P-FMNE cross-sections a, cross-sections of microneedle and microneedle tips (above/below scale bars: 10 μm/50 μm), the photograph in the red box on the left is an enlarged view of the area in the small red box at the tip of the microneedle. **b,** cross-sections of microneedle and the substrate (above/below scale bars: 10 μm/50 μm), the photograph in the red box on the left is an enlarged view of the area in the small red box at the base of the microneedle.

Supplementary Table 2 | PEDOT: PSS thickness at different heights.

Position	500 μm (The tip)	475 μm	450 μm	400 μm	300 μm	200 μm	100 μm	The substrate
Thickness of PEDOT:PSS	7.54 μm	3.79 μm	2.33 μm	2.58 μm	2.26 μm	2.82 μm	2.71 μm	2.79 μm

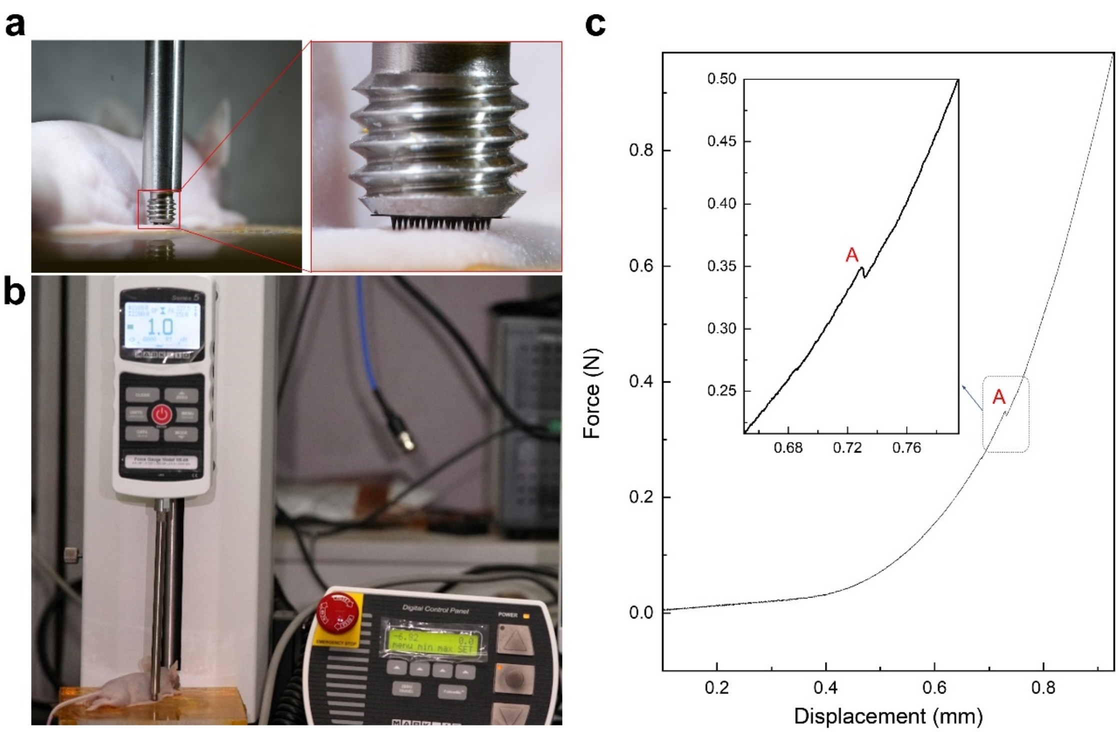

Supplementary Fig. 7 | Insertion force of the P-FMNE. a, photograph of the P-FMNE inserted into the skin of mouse, the photograph in the red box on the right is an enlarged view of the area in the small red box on the left (n = 3 mice). **b,** mechanical testing machine. **c,** force–displacement curve of the insertion process, the force–displacement curve on the left is an enlarged view of the area in the small blue dashed box on the right.

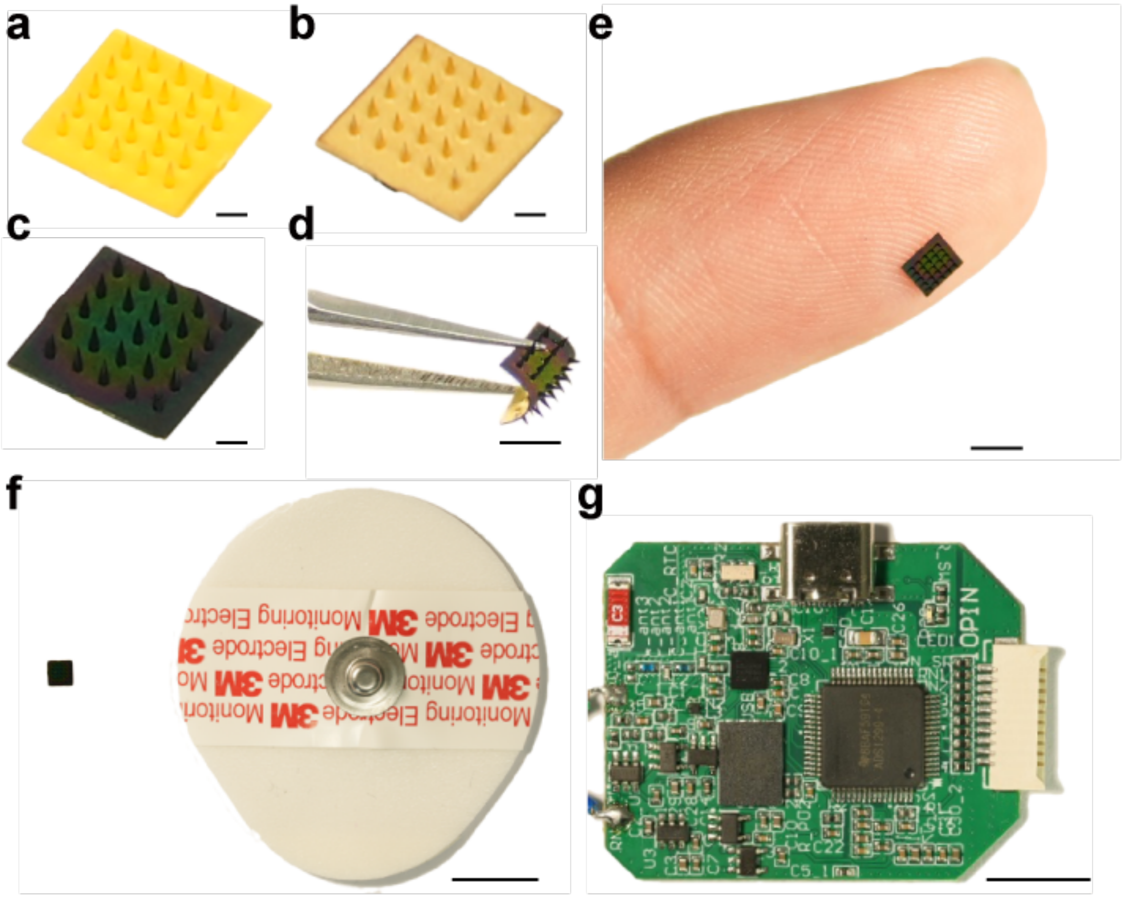

Supplementary Fig. 8 | Optical photographs of microneedle electrodes and PCB. a, Microneedle electrode before sputtering of Ti/Au (scale bar: 500 μm). **b,** microneedle electrode after sputtering Ti/Au (scale bar: 500 μm). **c,** microneedle electrode after electrodepositing PEDOT:PSS (scale bar: 500 μm). **d,** bent P-FMNE (scale bar: 3 mm). **e,** P-FMNE on an index finger for scale (scale bar: 3 mm). **f,** size comparison between P-FMNE and Ag/AgCl gel electrode (scale bar: 1 cm). **g,** electromyography (EMG) signal acquisition and wireless transmission circuit board (scale bar: 1 cm).

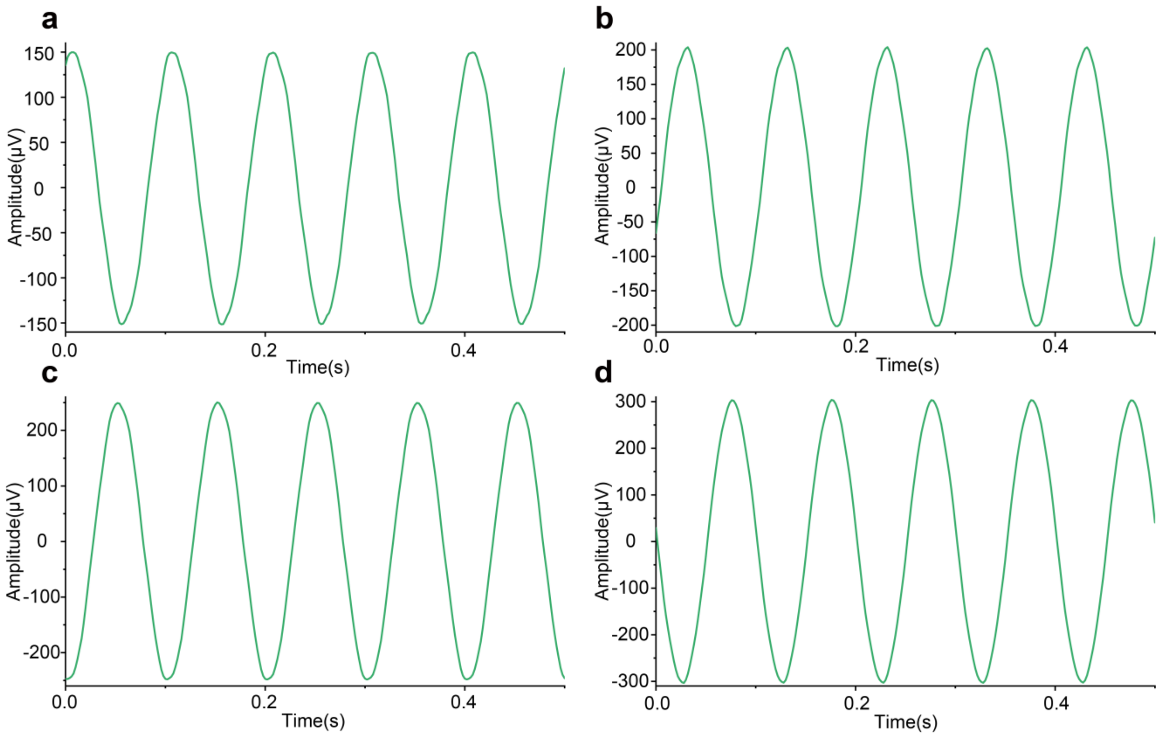

Supplementary Fig. 9 | Stimulator signal generator waveform acquired through wireless transmission module. a, generator outputs a 10 Hz sine signal with an amplitude of 150 μV. **b,** signal generator outputs a 10 Hz sine signal with an amplitude of 200 μV. **c,** signal generator outputs a 10 Hz sine signal with an amplitude of 250 μV. **d,** signal generator outputs a 10 Hz sine signal with an amplitude of 300 μV.

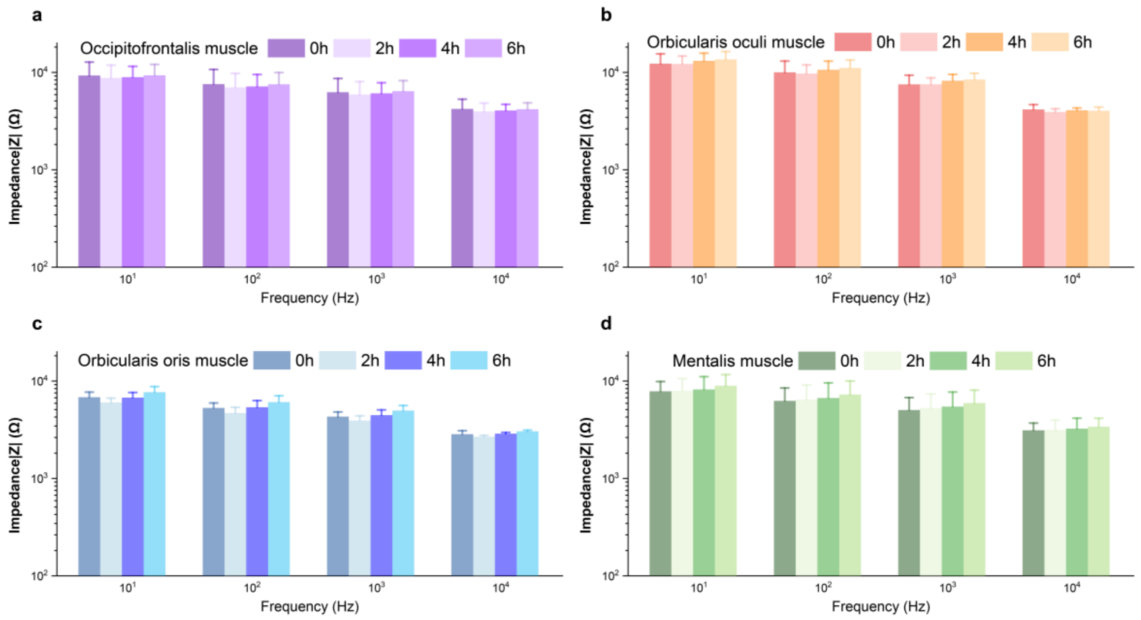

Supplementary Fig. 10 | Changes in EII at different muscles during 6 h of Biomask application. a, occipitofrontalis muscles. **b,** orbicularis oculi muscles. **c,** orbicularis oris muscles. **d,** mentalis muscles. All error bars denote S.D, n=3.

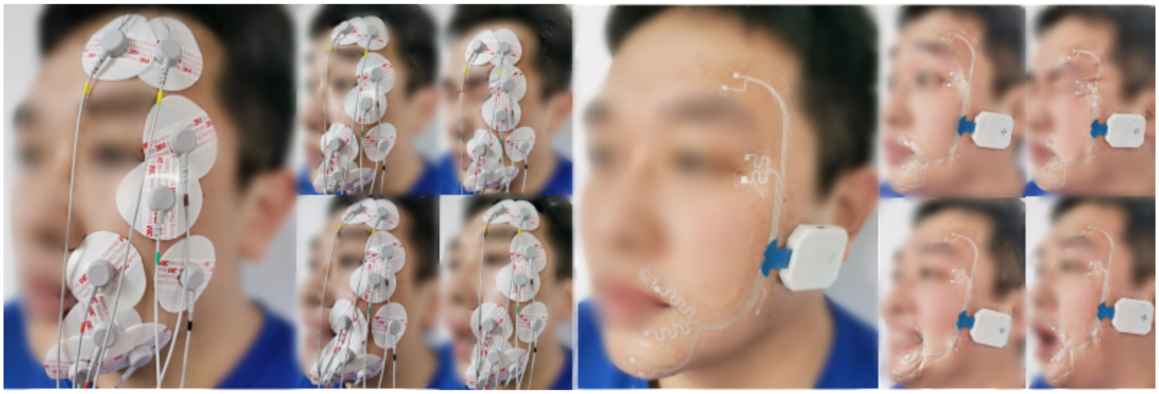

Supplementary Fig. 11 | Wearability of Ag/AgCl gel electrodes and Biomask when attached on the subject’s face, and the identifiable photograph is fully consented by the written consent.

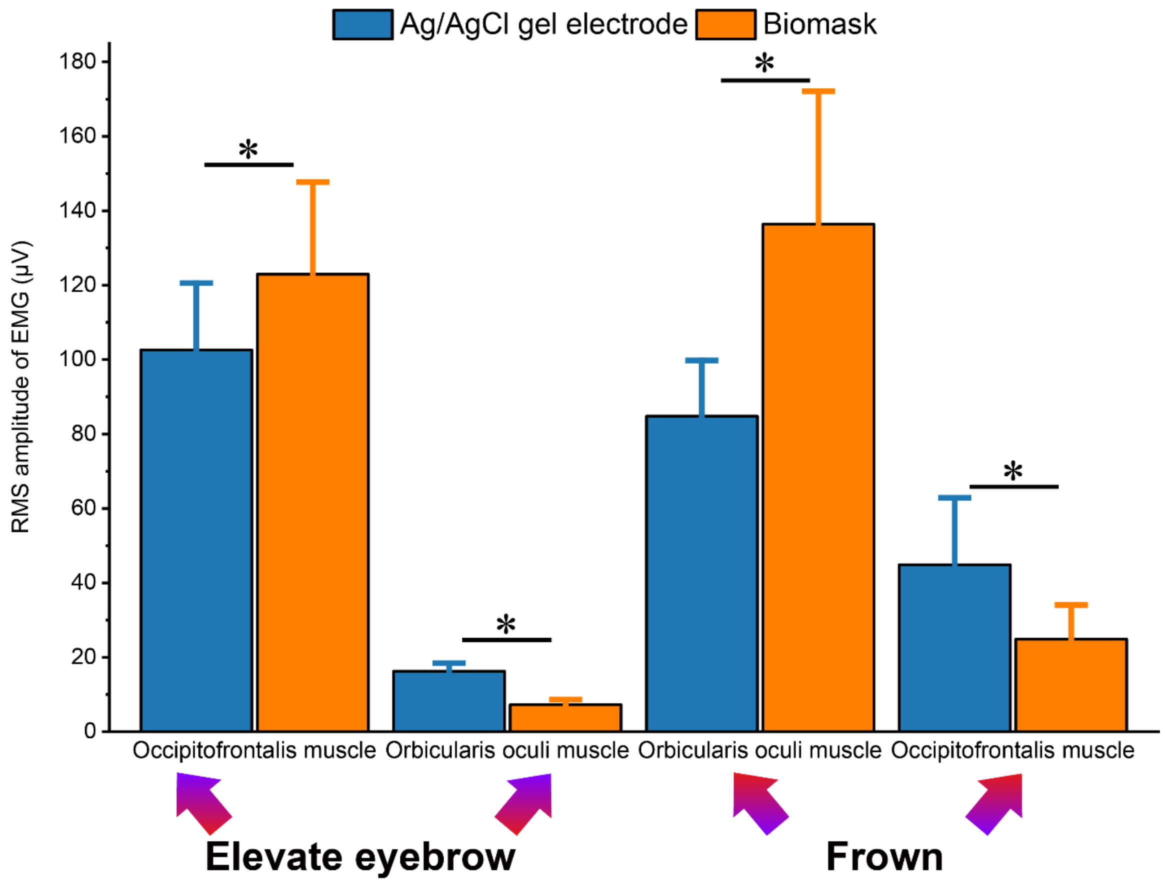

Supplementary Fig. 12 | Root mean square (RMS) amplitude of EMG for Ag/AgCl gel electrodes and Biomask electrodes at occipitofrontalis muscle and orbicularis oculi muscles. *P* values for comparing the RMS: Ag/AgCl gel electrode (n = 20) vs Biomask (n=20) of occipitofrontalis muscle in evaluating eyebrow, $P = 0.005$; Ag/AgCl gel electrode (n = 20) vs Biomask (n=20) of orbicularis oculi muscle in evaluating eyebrow, $P < 0.001$; Ag/AgCl gel electrode (n = 20) vs Biomask (n=20) of orbicularis oculi muscle in frowning, $P < 0.001$; Ag/AgCl gel electrode (n = 20) vs Biomask (n=20) of occipitofrontalis muscle in frowning, $P < 0.001$. All error bars denote s.d. *$P < 0.05$, and an unpaired, two-tailed student's t-test was used.

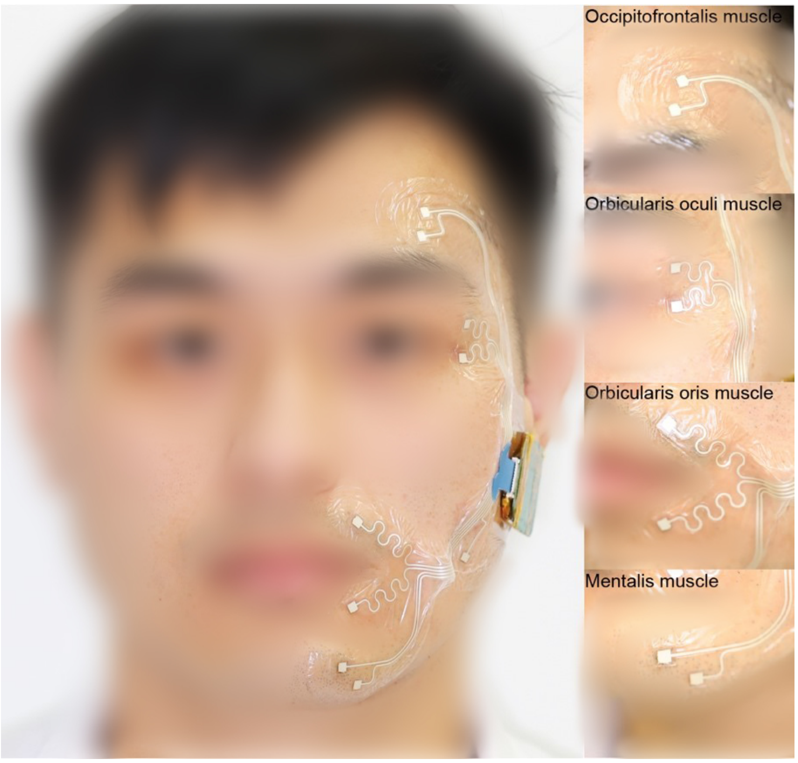

Supplementary Fig. 13 | Photograph of Biomask donned on the face of a healthy volunteer, and the identifiable photograph is fully consented by the written consent.

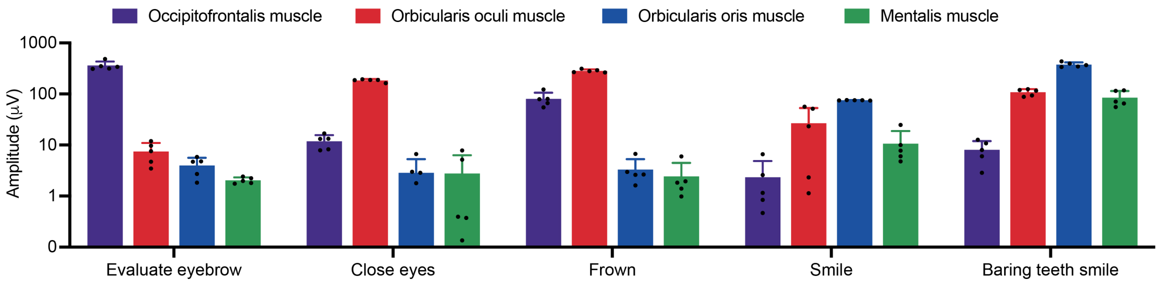

Supplementary Fig. 14 | Biomask records of the amplitude of EMG signals emanating from facial muscles in healthy volunteers during specific facial activities. All error bars denote s.d. (n=5)

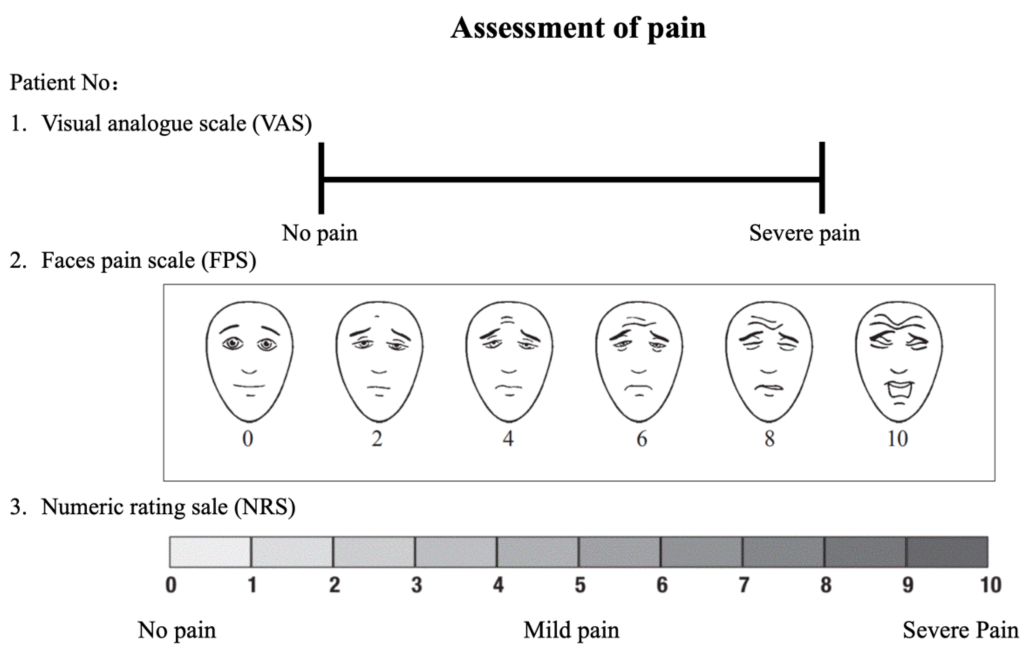

Supplementary Fig. 15 | Summary of pain assessment scale.

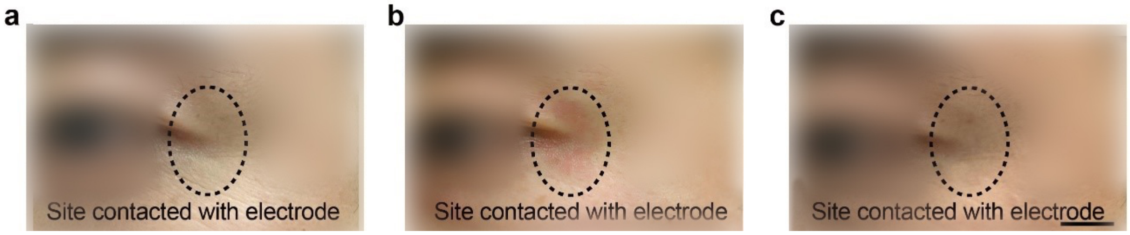

Supplementary Fig. 16 | Investigation into the Dermatological Impacts of Microneedle Electrode Applications. **a**, photo of skin before wearing the Biomask. **b**, photo of skin 0 min after removing the Biomask. **c,** photo of skin 30 mins after removing the Biomask. The black oval dashed line is the site contacted with electrode. The identifiable photograph is fully consented by the written consent.

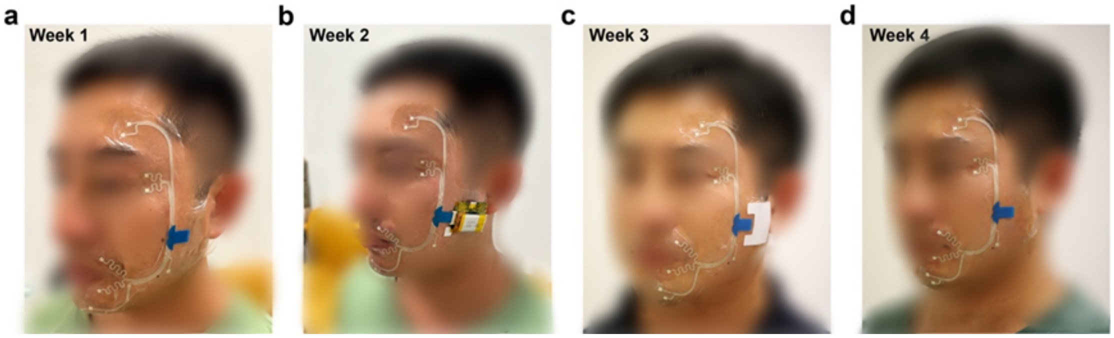

Supplementary Fig. 17 | Photos of Biomask wearing at distinct time points. a-d, photos of the same individual wearing the Biomask at week-1, week-2, week-3, and week-4 intervals. The integrated design ensures precise electrode positioning with each application. The identifiable photograph is fully consented by the written consent.

Supplementary Video 1 | Video of Biomask being used to thoroughly evaluate facial muscle functionality while patient performs a sequence of four distinct movements, and the identifiable photograph is fully consented by the written consent.

Supplementary Video 2 | Video of Biomask monitoring direct facial nerve electrical stimulation for identifying and assessing nerve–muscle interfaces, compared to the most advanced commercially available intraoperative electrophysiological monitoring equipment.

Supplementary Video 3 | Video of Biomask monitoring of intraoperative passive tugging on the facial nerve induced by surgical manipulation, compared to the most advanced commercially available intraoperative electrophysiological monitoring equipment.

Supplementary Video 4 | Video of Biomask monitoring healthy side of facial muscle functionality in a vestibular schwannoma patient following tumor resection.

Supplementary Video 5 | Video of Biomask monitoring affected side of facial muscle functionality in a vestibular schwannoma patient following tumor resection.
