# Supplementary material for: Wireless facial biosensing system for monitoring facial palsy with flexible microneedle electrode arrays
Source: NPJ Digit Med. 2024 Jan 15;7:13. doi: 10.1038/s41746-024-01002-1 (PMC10789865; doi:10.1038/s41746-024-01002-1)
Supplement: Supplementary file 2 — nr-reporting-summary [file 41746_2024_1002_MOESM2_ESM.pdf]

nature portfolio

Corresponding author(s): Dong Huang; Zhihong Li; Wang Jia

Last updated by author(s): Dec 11, 2023

Reporting Summary

Nature Portfolio wishes to improve the reproducibility of the work that we publish. This form provides structure for consistency and transparency in reporting. For further information on Nature Portfolio policies, see our Editorial Policies and the Editorial Policy Checklist.

Statistics

For all statistical analyses, confirm that the following items are present in the figure legend, table legend, main text, or Methods section.

n/a	Confirmed	
☐	☒	The exact sample size (*n*) for each experimental group/condition, given as a discrete number and unit of measurement
☐	☒	A statement on whether measurements were taken from distinct samples or whether the same sample was measured repeatedly
☐	☒	The statistical test(s) used AND whether they are one- or two-sided<br>*Only common tests should be described solely by name; describe more complex techniques in the Methods section.*
☒	☐	A description of all covariates tested
☒	☐	A description of any assumptions or corrections, such as tests of normality and adjustment for multiple comparisons
☐	☒	A full description of the statistical parameters including central tendency (e.g. means) or other basic estimates (e.g. regression coefficient) AND variation (e.g. standard deviation) or associated estimates of uncertainty (e.g. confidence intervals)
☐	☒	For null hypothesis testing, the test statistic (e.g. *F*, *t*, *r*) with confidence intervals, effect sizes, degrees of freedom and *P* value noted<br>*Give P values as exact values whenever suitable.*
☒	☐	For Bayesian analysis, information on the choice of priors and Markov chain Monte Carlo settings
☒	☐	For hierarchical and complex designs, identification of the appropriate level for tests and full reporting of outcomes
☒	☐	Estimates of effect sizes (e.g. Cohen's *d*, Pearson's *r*), indicating how they were calculated

Our web collection on statistics for biologists contains articles on many of the points above.

Software and code

Policy information about availability of computer code

Data collection	Facial EMG signals were amplified and analog-to-digital converted using a circuit module based on ADS1299-4 and transmitted to a tablet using a Bluetooth transmission module based on nRF52840. The EMG signal visualization software developed by SDK 30 was installed on the tablet and the EMG signals were stored in the form of BDF files.
Data analysis	Data analysis was conducted by using OriginPro 2022 (SR1 v.9.9.0.225), GraphPad (Prism 8.4.3) and Microsoft Excel 2016 (v.1807, 10325.20082) .

For manuscripts utilizing custom algorithms or software that are central to the research but not yet described in published literature, software must be made available to editors and reviewers. We strongly encourage code deposition in a community repository (e.g. GitHub). See the Nature Portfolio guidelines for submitting code & software for further information.

Data

Policy information about availability of data

All manuscripts must include a data availability statement. This statement should provide the following information, where applicable:

- Accession codes, unique identifiers, or web links for publicly available datasets
- A description of any restrictions on data availability
- For clinical datasets or third party data, please ensure that the statement adheres to our policy

All data supporting the findings of this study are available within the paper and its Supplementary Information.

Research involving human participants, their data, or biological material

Policy information about studies with human participants or human data. See also policy information about sex, gender (identity/presentation), and sexual orientation and race, ethnicity and racism.

Reporting on sex and gender	Sex and gender were not canisdered in the study design, and the recruited population characteristics were evenly split across males and females. Sex and/or gender data was collected from patients self reporting this data. There was no sex/gender based analysis performed because facial paralysis is a common issue affecting all sexes and genders.
Reporting on race, ethnicity, or other socially relevant groupings	Socially constructed and socially relevant categorization variable were not involved in this study.
Population characteristics	Population characteristics for patients: n=6 (Age range: 24-43), 3 male and 3 female, mean age= 32.3, Diagnosis: vestibular schwannoma (left/right), Surgery: retrosigmoid approach tumor resection.
Recruitment	Patients were recruited from existing patient populations at Beijing Tiantan Hospital, Capital Medical University.
Ethics oversight	Beijing Tiantan Hospital, Capital Medical University.

Note that full information on the approval of the study protocol must also be provided in the manuscript.

Field-specific reporting

Please select the one below that is the best fit for your research. If you are not sure, read the appropriate sections before making your selection.

☒ Life sciences ☐ Behavioural & social sciences ☐ Ecological, evolutionary & environmental sciences

For a reference copy of the document with all sections, see nature.com/documents/nr-reporting-summary-flat.pdf

Life sciences study design

All studies must disclose on these points even when the disclosure is negative.

Sample size	This work represents an early stage pilot study and as such,sample size calculations were not performed. The sample size(n=6)was determined by patient availability.
Data exclusions	No data were excluded from any of the analyses.
Replication	All experimental findings, including material characterization, device fabrication, animal experiments and clinical trial were reliably reproduced. On body measurement trends (EMG signals) were measured across multiple healthy subjects as well as patients.
Randomization	The focus of this study was to demonstrate the clinical feasibility of the Biomask system for facial paralysis Monitoring, and as such randomization was not relevant to this study. Participants were recruited without any age,gender,or racial bias.
Blinding	Anonymous IDs were assigned to all study subjects to protect their privacy. No blinding was conducted, as it does not impact the facial paralysis Monitoring.

Behavioural & social sciences study design

All studies must disclose on these points even when the disclosure is negative.

Study description	
Research sample	
Sampling strategy	
Data collection	
Timing	
Data exclusions	
Non-participation	

Randomization

Ecological, evolutionary & environmental sciences study design

All studies must disclose on these points even when the disclosure is negative.

Study description

Research sample

Sampling strategy

Data collection

Timing and spatial scale

Data exclusions

Reproducibility

Randomization

Blinding

Did the study involve field work? ☐ Yes ☐ No

Field work, collection and transport

Field conditions

Location

Access & import/export

Disturbance

Reporting for specific materials, systems and methods

We require information from authors about some types of materials, experimental systems and methods used in many studies. Here, indicate whether each material, system or method listed is relevant to your study. If you are not sure if a list item applies to your research, read the appropriate section before selecting a response.

Materials & experimental systems

n/a	Involved in the study	
☒	☐	Antibodies
☒	☐	Eukaryotic cell lines
☒	☐	Palaeontology and archaeology
☐	☒	Animals and other organisms
☐	☒	Clinical data
☒	☐	Dual use research of concern
☒	☐	Plants

Methods

n/a	Involved in the study	
☒	☐	ChIP-seq
☒	☐	Flow cytometry
☒	☐	MRI-based neuroimaging

Antibodies

Antibodies used

Validation

Eukaryotic cell lines

Policy information about cell lines and Sex and Gender in Research

Cell line source(s)	
Authentication	
Mycoplasma contamination	
Commonly misidentified lines (See ICLAC register)	

Palaeontology and Archaeology

Specimen provenance	
Specimen deposition	
Dating methods	

☐ Tick this box to confirm that the raw and calibrated dates are available in the paper or in Supplementary Information.

Ethics oversight	

Note that full information on the approval of the study protocol must also be provided in the manuscript.

Animals and other research organisms

Policy information about studies involving animals; ARRIVE guidelines recommended for reporting animal research, and Sex and Gender in Research

Laboratory animals	6-8 weeks old male BALB/c Nude mice raised in a Specific Pathogen Free (SPF) environment were used in the study.
Wild animals	The study did not involve wild animals.
Reporting on sex	Sex was not considered in the study.
Field-collected samples	The study did not involve samples collected from the field.
Ethics oversight	All animal procedures were performed following the guidelines of Public Health Service Policy on Humane Care of Laboratory Animals and approved by the Institutional Animal Care and Use Committee of the Beijing Tiantan Hospital, Capital Medical University.

Note that full information on the approval of the study protocol must also be provided in the manuscript.

Clinical data

Policy information about clinical studies

All manuscripts should comply with the ICMJE guidelines for publication of clinical research and a completed CONSORT checklist must be included with all submissions.

Clinical trial registration	This study was approved by the Beijing Tiantan Hospital, Capital Medical University Investigational Review Board, and informed consent was obtained from all patients prior to surgery and research participation. (HX-B-2022025; May 27, 2022). This study was also approved by Chinese Clinical Trial Registry (ChiCTR) with the trial registration number (ChiCTR2300072887; June 27, 2023). All animal procedures were performed in accordance with the guidelines of the Public Health Service Policy on Humane Care of Laboratory Animals and approved by the Institutional Animal Care and Use Committee of Beijing Tiantan Hospital, Capital Medical University (202101001).
Study protocol	Trial details can be available within the paper and its Supplementary Information.
Data collection	Prior to Biomask application, the facial cleanliness of patients was ensured and traditional electrodes were positioned , and performed the specified actions, repeating each three times. The EMG data from distinct facial muscles during these diverse actions were collected for subsequent analyses. For intraoperative neurophysiological monitoring, the Biomask was placed on the facial skin overlying the pertinent muscles of the patient and concurrently monitored using standard needle EMG techniques. Nerve–muscle activity was recorded once the target nerves were surgically exposed, and direct current stimulation was applied under the guidance and supervision of the lead surgeon. The data collection took place from June 24, 2022 to June 30, 2023.

Outcomes	The primary variables in this study were pre- and post-operation and healthy and affected side EMG signal changes in patients with facial paralysis. Recovery from facial paralysis can be measured by the amplitude of the EMG signal.

Dual use research of concern

Policy information about dual use research of concern

Hazards

Could the accidental, deliberate or reckless misuse of agents or technologies generated in the work, or the application of information presented in the manuscript, pose a threat to:

No	Yes	
☐	☐	Public health
☐	☐	National security
☐	☐	Crops and/or livestock
☐	☐	Ecosystems
☐	☐	Any other significant area

Experiments of concern

Does the work involve any of these experiments of concern:

No	Yes	
☐	☐	Demonstrate how to render a vaccine ineffective
☐	☐	Confer resistance to therapeutically useful antibiotics or antiviral agents
☐	☐	Enhance the virulence of a pathogen or render a nonpathogen virulent
☐	☐	Increase transmissibility of a pathogen
☐	☐	Alter the host range of a pathogen
☐	☐	Enable evasion of diagnostic/detection modalities
☐	☐	Enable the weaponization of a biological agent or toxin
☐	☐	Any other potentially harmful combination of experiments and agents

Plants

Seed stocks	The study did not involve plants.
Novel plant genotypes	The study did not involve plants.
Authentication	The study did not involve plants.

ChIP-seq

Data deposition

☐ Confirm that both raw and final processed data have been deposited in a public database such as GEO.

☐ Confirm that you have deposited or provided access to graph files (e.g. BED files) for the called peaks.

Data access links<br>*May remain private before publication.*	
Files in database submission	
Genome browser session<br>(e.g. UCSC)	

Methodology

Replicates

Sequencing depth

Antibodies

Peak calling parameters

Data quality

Software

Flow Cytometry

Plots

Confirm that:

- [ ] The axis labels state the marker and fluorochrome used (e.g. CD4-FITC).
- [ ] The axis scales are clearly visible. Include numbers along axes only for bottom left plot of group (a 'group' is an analysis of identical markers).
- [ ] All plots are contour plots with outliers or pseudocolor plots.
- [ ] A numerical value for number of cells or percentage (with statistics) is provided.

Methodology

Sample preparation

Instrument

Software

Cell population abundance

Gating strategy

- [ ] Tick this box to confirm that a figure exemplifying the gating strategy is provided in the Supplementary Information.

Magnetic resonance imaging

Experimental design

Design type

Design specifications

Behavioral performance measures

Acquisition

Imaging type(s)

Field strength

Sequence & imaging parameters

Area of acquisition

Diffusion MRI ☐ Used ☐ Not used

Preprocessing

Preprocessing software

Normalization

Normalization template

Noise and artifact removal

Volume censoring

Statistical modeling & inference

Model type and settings

Effect(s) tested

Specify type of analysis: ☐ Whole brain ☐ ROI-based ☐ Both

Statistic type for inference

(See Eklund et al. 2016)

Correction

Models & analysis

n/a	Involved in the study
☐	☐ Functional and/or effective connectivity
☐	☐ Graph analysis
☐	☐ Multivariate modeling or predictive analysis

Functional and/or effective connectivity

Graph analysis

Multivariate modeling and predictive analysis
